# HDAC3 as an Immunometabolic Rheostat: Molecular Mechanisms of Deacylation Plasticity, Lactylation Dynamics, and Spatiotemporal Regulation

**DOI:** 10.3390/biom16070980

**Published:** 2026-07-03

**Authors:** Yifan Bu, Wanying Li, Songzhe Li, Zhihua Hao, Baiyang Gu, Jing Chen

**Affiliations:** College of Basic Medical Sciences, Heilongjiang University of Chinese Medicine, Harbin 150040, China; b8068691@163.com (Y.B.);

**Keywords:** HDAC3, immunometabolic reprogramming, lysine lactylation, catalytic redistribution, delactylation

## Abstract

Histone deacetylase 3 (HDAC3) is a key node linking immunometabolism, chromatin regulation, and inflammatory transcriptional programs. Rather than functioning simply as a nuclear deacetylase, HDAC3 output is jointly shaped by corepressor-complex assembly, metabolic and acyl-substrate availability, and compartment-specific substrate access. The identification of lysine lactylation and the discovery of delactylase activity in HDAC1–3 have expanded the mechanistic boundaries of HDAC3, repositioning it from a canonical deacetylase toward an emerging regulatory node involved in the dynamic control of multiple acyl modifications. This review examines the complex-dependent activation of HDAC3, its regulation of nuclear inflammatory transcriptional thresholds, the proposed redistribution of its catalytic output across acetylated and lactylated substrates under increased lactate load, and candidate extra-nuclear non-histone acylation networks involving inflammatory signaling proteins and metabolic enzymes. Current evidence supports bona fide delactylase activity of HDAC3 in biochemical systems; however, whether HDAC3 directly delactylates specific cytoplasmic substrates in physiologically relevant settings requires further validation at the compartmental, site-specific, and functional levels. Viewing HDAC3 as an immunometabolic rheostat helps explain its context-dependent functions in inflammatory homeostasis, acute activation, and metabolic stress, and provides a conceptual basis for developing selective, complex-state-sensitive, and function-stratified HDAC3-targeted strategies.

## 1. Introduction

Immune responses are not simply linear processes determined by receptor recognition and signaling cascades. Rather, they are dynamic systems jointly shaped by metabolic state, chromatin plasticity, and transcriptional thresholds. During quiescence, activation, differentiation, and effector execution, immune cells undergo highly ordered metabolic reprogramming. These metabolic changes not only provide energy and biosynthetic precursors, but also feedback on gene expression and immune phenotypes through changes in metabolite abundance, cofactor supply, and enzymatic activity [1,2]. Metabolism should therefore no longer be viewed merely as the background condition of immune responses, but as an important regulatory layer that conveys information about cellular state, modulates inflammatory intensity, and influences disease outcomes.

Lactate is one of the most representative signaling metabolites in immunometabolism research. Traditionally, lactate has often been regarded as a metabolic end product of enhanced glycolysis. However, accumulating evidence indicates that lactate can directly participate in shaping the immune microenvironment and regulating transcriptional programs. In highly glycolytic settings, lactate can influence macrophage polarization and promote phenotypes associated with immunosuppression or tissue remodeling [3]. At the same time, lactate can inhibit histone deacetylase (HDAC) activity and induce changes in gene expression, thereby creating an observable link among glycolytic flux, deacylation capacity, and transcriptional state [4]. These findings suggest that lactate is not simply a metabolic waste product, but a molecular signal capable of translating metabolic pressure into changes in immune phenotype.

The concept of histone lysine lactylation (Kla) has further reshaped the framework of this field. Enhanced glycolysis and lactate accumulation can promote Kla formation, which is associated with specific gene expression programs, thereby providing a more direct molecular route linking lactate, chromatin modification, and transcriptional regulation [5]. Subsequent studies demonstrated that class I HDACs, especially HDAC1–3, possess delactylase activity, indicating that Kla is not an irreversible metabolic deposit but an epigenetic modification subject to dynamic enzymatic regulation [6]. Accordingly, a central question in immunometabolism has shifted: lactate may not only influence HDAC activity, but may also reshape the timing and direction of inflammatory responses by altering the balance between the writing and erasing of multiple lysine acylation marks.

Although HDAC1–3 all exhibit substantial delactylase activity, the focus of this review on HDAC3 does not imply that HDAC3 is universally dominant over HDAC1 or HDAC2. HDAC1 and HDAC2 function as closely related catalytic subunits within several complexes, including Sin3, NuRD, CoREST, and MiDAC, whose distinct architectures may shape substrate accessibility and site selectivity. By contrast, HDAC3 provides a particularly informative model because its catalytic output is tightly integrated with NCoR/SMRT-DAD-dependent licensing, inositol-phosphate-mediated stabilization, inflammatory transcriptional control, and spatial redistribution. HDAC3 is not a deacetylase whose function is determined simply by its expression level. Its effective catalytic activity depends on licensing by the nuclear receptor corepressor/silencing mediator for retinoid and thyroid hormone receptors (NCoR/SMRT) complex and its associated structural domains, and is further regulated conformationally by inositol phosphates [7,8]. This complex dependence gives HDAC3 a context-sensitive regulatory architecture, allowing its functional output to vary with metabolic state, complex assembly, and subcellular localization.

The functions of HDAC3 in immune cells also display clear bidirectional features. Studies in macrophages have shown that HDAC3 can both support lipopolysaccharide (LPS)-induced inflammatory gene programs and restrain interleukin-4 (IL-4)-driven alternative activation [9,10]. In addition, some physiological functions of HDAC3 do not depend entirely on its deacetylase activity, but are closely related to its complex interactions and scaffold-like roles [11]. Together, these findings indicate that HDAC3 cannot be simply categorized as either a pro-inflammatory or an anti-inflammatory factor, nor should it be understood as a transcriptional repressor acting in only one direction. A more appropriate view is that HDAC3 functions as an epigenetic regulatory node whose activity is jointly shaped by complex assembly, metabolic context, and substrate spectrum, thereby altering inflammatory transcriptional thresholds and cell-fate decisions across different immune states.

Research on HDAC3 now extends beyond nuclear chromatin regulation to spatial and substrate dimensions. Existing studies suggest that HDAC3 can localize outside the nucleus and participate in membrane-associated or cytoplasmic signaling events [12]. At the same time, the continuing discovery of non-histone lactylation and other short-chain acyl modifications has made the potential substrate environment of HDAC3 increasingly complex. Under conditions of high lactate load, how HDAC3 allocates its catalytic capacity among acetylated, lactylated, and other acylated substrates, whether metabolic pressure can drive a reallocation of its cellular deacylation flux, and whether such changes are sufficient to influence the phase-specific transition of inflammatory programs remain insufficiently answered questions in the field.

**Figure 1:**
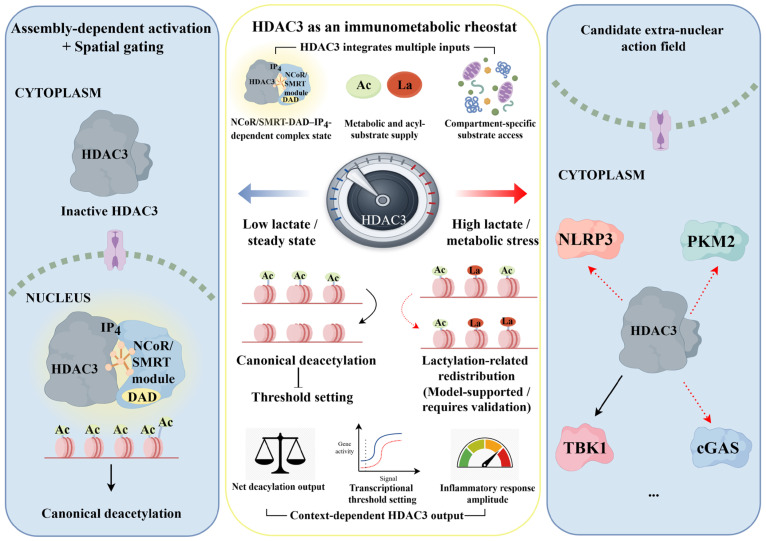
Conceptual framework of HDAC3 as an immunometabolic rheostat. Note: HDAC3, histone deacetylase 3; NCoR, nuclear receptor corepressor; SMRT, silencing mediator for retinoid and thyroid hormone receptors; DAD, deacetylase activation domain; IP_4_, inositol 1,4,5,6-tetrakisphosphate; Ac, acetylation; La, lactylation; TBK1, TANK-binding kinase 1; cGAS, cyclic GMP-AMP synthase; NLRP3, NLR family pyrin domain containing 3; PKM2, pyruvate kinase M2. Solid arrows indicate experimentally supported activities or established relationships, whereas red dotted arrows indicate candidate HDAC3-linked extra-nuclear mechanisms that require direct validation. Colors are used to distinguish different functional modules and do not indicate evidence strength. The figure summarizes how complex state, acyl-substrate supply, and compartment-specific substrate access shape the context-dependent nuclear and extra-nuclear outputs of HDAC3. Created with Figdraw (ID: YIAWY88ee4).

In light of these advances, HDAC3 can be viewed as an immunometabolic rheostat situated at the intersection of metabolic and epigenetic regulation. Here, the term “rheostat” denotes a context-dependent regulatory system in which HDAC3 output is shaped by three principal inputs: NCoR/SMRT-DAD–IP_4_-dependent complex state, metabolic and acyl-substrate availability, and compartment-specific substrate access. These interacting inputs modulate net deacylation output, transcriptional thresholds, and inflammatory transcriptional programs. The metaphor is qualitative and does not imply a single linear or quantitatively calibrated control variable, nor does it assume uniform competition between acetylated and lactylated substrates. This framework moves beyond binary interpretations of HDAC3 as either pro-inflammatory or anti-inflammatory, or as exclusively a deacetylase or delactylase, and instead places it within a dynamic regulatory network that more closely reflects physiological cellular states. Accordingly, systematic consideration of the structural dependence of HDAC3, its nuclear transcriptional functions, lactylation-related deacylation potential, extra-nuclear functional boundaries, and pharmacological targeting strategies may clarify its mechanistic position in immunometabolic reprogramming and provide a conceptual basis for more selective HDAC3 regulation. Figure 1 summarizes this framework by linking assembly-dependent activation and spatial gating to metabolism-sensitive catalytic output and candidate extra-nuclear action fields.

## 2. Structural Basis: Complex-Dependent Activation and Spatial Compartmentalization

HDAC3 is best viewed as an assembly-dependent enzymatic module rather than a constitutively active deacetylase. Its stable catalytic activity requires assembly with the NCoR/SMRT deacetylase activation domain and stabilization of this interface by inositol polyphosphates. These dependencies create an adjustable activity threshold through which complex assembly, interface-stabilizing factors, and subcellular partitioning tune HDAC3-dependent transcriptional regulation across different signaling and metabolic states.

### 2.1. Catalytic Core and Domain Logic: The Molecular Premise of Assembly Dependence

HDAC3 belongs to the class I histone deacetylase family and contains a highly conserved deacetylase domain (DAC domain), but it also possesses a non-conserved C-terminal tail region that is clearly distinct from those of its homologs HDAC1, HDAC2, and HDAC8 [13]. Crystal structure analyses have shown that the catalytic core of HDAC3 contains an active pocket built around an Asp-His charge-relay system and a zinc ion (Zn^2+^), which provides the chemical potential to hydrolyze the amide bond of acetylated lysine [8]. However, from a cellular functional perspective, the presence of a catalytic pocket does not necessarily mean the formation of stable enzymatic activity.

A key feature of HDAC3 is that, in its free state, it is not a fully activated and highly efficient catalytic molecule. Free HDAC3 adopts a highly dynamic and unstable conformation in solution, and its active pocket remains in a “self-inhibited” or “collapsed” state, with very low substrate affinity. It requires participation of the corepressor complex to achieve a more stable conformation and more effective substrate processing [7,8]. This corepressor dependence restricts indiscriminate HDAC3 activity and provides a structural basis for separating its catalytic and scaffold functions. On the one hand, it prevents indiscriminate enzymatic activity by HDAC3 under non-specific conditions and thereby avoids excessive genome-wide deacetylation. On the other hand, this conformational plasticity allows HDAC3 to engage different interacting proteins and exert scaffold functions independent of catalysis, thereby switching between nuclear transcriptional repression and extra-nuclear signal transduction [11,14]. HDAC3 should therefore not be viewed as a single catalytic enzyme, but rather as a multilayered regulatory node composed of a catalytic domain, complex interfaces, and spatial control modules.

This framework also lays the foundation for the later discussion of lactylation-related deacylation. The hydrophobic tunnel within the HDAC3 catalytic pocket has a certain degree of extensibility, which provides structural plausibility for accommodating acyl modifications bulkier than an acetyl group, such as lactyl and crotonyl groups, under specific metabolic pressure. This forms the structural basis for considering HDAC3 a potentially multifunctional deacylase [6,15,16,17]. However, how this potential is translated into actual substrate choice in cells still cannot be understood apart from complex state and the local substrate environment. A more cautious conclusion is that HDAC3 possesses the structural and enzymatic basis for handling multiple acyl substrates, but its actual output is likely determined jointly by complex assembly, substrate accessibility, and spatial distribution.

### 2.2. DAD-Dependent Activation: Enzymatic Licensing Mediated by the NCoR/SMRT Complex

The highly conserved deacetylase activation domain (DAD) of NCoR/SMRT is the core structural unit that underlies the complex-dependent activation of HDAC3. NCoR/SMRT does not merely act as a “carrier” for HDAC3; rather, its DAD itself is an essential activating factor required for robust HDAC3 enzymatic activity. Disruption of the DAD-HDAC3 interaction leads to a marked reduction or loss of HDAC3-associated deacetylase activity [7]. This means that the enzymatic switch of HDAC3 is effectively controlled by the corepressor, and whether DAD assembly occurs is itself a rate-limiting step that can be regulated by cellular signaling. Only when HDAC3 forms a stable interface with DAD can its catalytic core more readily adopt a conformation suitable for substrate binding and catalysis.

Structural studies have further clarified this activation mechanism. The SMRT-DAD contains finely organized functional modules: its N-terminal DAD-specific motif and C-terminal SANT-like domain together form the activation interface. One SANT motif induces the active conformation of HDAC3, whereas another forms a histone interaction domain (HID) that enhances histone substrate affinity and delivery efficiency [18,19]. This complex assembly process ensures that the catalytic activity of HDAC3 is not released indiscriminately, but is instead directed toward specific chromatin regions. The dual-module logic of activation plus substrate delivery forms a positive functional loop, ensuring that once HDAC3 is assembled, it can sustain efficient local deacetylation on chromatin. Thus, the regulation of HDAC3 by NCoR/SMRT is not simply a matter of “switching on enzymatic activity”, but simultaneously accomplishes conformational stabilization, substrate delivery, and chromatin targeting.

In this process, inositol tetraphosphate, particularly Ins(1,4,5,6)P_4_, acts as a critical “molecular glue”. Structural evidence shows that IP_4_ is embedded in the charged interface between HDAC3 and DAD, where it locks the complex into an active conformation through electrostatic interactions [8]. Moreover, regulation of class I HDAC complexes by inositol phosphates may not be unique to HDAC3. Studies suggest that class I HDACs broadly share regulatory mechanisms involving inositol phosphates [20], pointing to a more general form of complex allosteric control. This means that HDAC3 enzymatic activity depends not only on protein–protein interactions, but also on the local small-molecule environment and interface chemistry.

This DAD/IP_4_ dependence gives HDAC3 an intrinsic regulatory threshold. By altering NCoR/SMRT complex assembly, DAD interface stability, inositol phosphate availability, and chromatin recruitment, cells can tune the actual output of HDAC3 across different genomic regions. This mechanism helps explain why HDAC3 can function as the catalytic core of a corepressor complex under homeostatic conditions to maintain low transcriptional noise, yet be redeployed into distinct transcriptional programs in the context of inflammation or metabolic reprogramming. In other words, the catalytic activity of HDAC3 is better understood as an assembly-state function licensed by the corepressor complex, rather than as an inherent output of the free enzyme itself.

### 2.3. Spatial Dynamics: Nucleocytoplasmic Shuttling and the Reshaping of Functional Compartmentalization

Although HDAC3 is often discussed in the context of nuclear transcription, it does not statically reside on chromatin. Instead, it displays marked plasticity in subcellular distribution, and this spatial distribution defines its functional boundaries. Available evidence indicates that the spatial dynamics of HDAC3 are constrained at least by a three-part mechanism involving intrinsic trafficking signals, partner-protein anchoring, and signal-dependent modifications. This allows cells to reshape the balance between nuclear transcriptional gating and extra-nuclear non-genomic networks according to metabolic and inflammatory status.

Domain mapping and functional dissection studies suggest that the middle region of HDAC3 contains sequence information that promotes extra-nuclear distribution or nuclear export, whereas the C-terminal region is closely associated with nuclear enrichment and nuclear function [21]. Functional separation experiments have shown that the nucleocytoplasmic distribution of HDAC3 is not driven by passive diffusion. Retention of nuclear localization information can drive HDAC3 into the nucleus and maintain its repressive effect at promoter regions, whereas deletion of the corresponding region favors cytoplasmic retention and weakens transcriptional regulatory capacity [22]. Although different studies vary in their definition of the exact boundaries, they consistently indicate that the subcellular distribution of HDAC3 is not purely a passive process, but is jointly controlled by localization signals, protein interactions, and cellular state. Spatial distribution thus becomes an important prerequisite for HDAC3 functional selection: the same molecule can access different substrates and regulatory networks depending on whether it resides in the nucleus, the cytoplasm, or membrane-associated regions.

Within the nucleus, HDAC3 primarily participates in chromatin deacetylation, transcriptional repression, and the setting of gene-expression thresholds through the NCoR/SMRT complex [7,8]. This function depends not only on complex assembly, but also on recruitment of HDAC3 to specific promoters, enhancers, or nuclear receptor-regulated regions. Nuclear HDAC3 does not simply shut transcription off. Rather, by modulating local acetylation levels and the stability of the corepressor complex, it determines whether certain genes maintain low basal expression in the resting state and whether repression can be rapidly relieved upon stimulation.

The nucleocytoplasmic shuttling of HDAC3 can be jointly regulated by inflammatory signaling and interactions with chaperone-like proteins. Under tumor necrosis factor alpha (TNF-α) stimulation, for example, HDAC3 can form a complex with inhibitor of κB alpha (IκBα) in the cytoplasm. Once TNF-α induces IκBα degradation, HDAC3 translocates into the nucleus, accompanied by increased nuclear HDAC3 activity, thereby suppressing the transcription of metabolic genes such as peroxisome proliferator-activated receptor gamma (PPARγ) [23]. This indicates that within the TNF-α signaling pathway, IκBα functions not only as an inhibitor of NF-κB, but also as a cytoplasmic anchoring protein for HDAC3, directly reshaping its effective field of action through protein interaction.

Beyond classical nuclear-cytoplasmic shuttling, HDAC3 can undergo more distal spatial redirection under specific conditions. HDAC3 has been shown to localize at the plasma membrane and to act as a substrate of Src kinase, suggesting that it can enter membrane-associated signaling events and gain spatial access to non-classical extra-nuclear substrates [12]. In addition, HDAC3 has been reported to possess a deacetylase-independent “molecular chaperone” function, for example by facilitating the transport of phosphorylated testicular receptor 2 (TR2) from the cytoplasm to specific nuclear domains [24]. These studies suggest that the functional boundary of HDAC3 is not defined solely by catalytic activity. In different cellular locations, it may produce even opposing functional outputs through distinct substrate sets and interaction networks.

**Figure 2:**
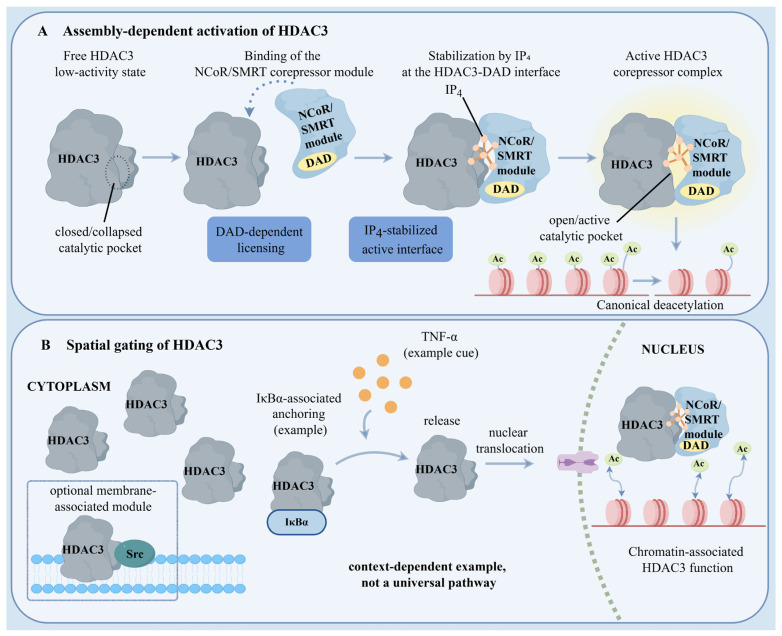
Molecular licensing and spatial redistribution of HDAC3. Note: HDAC3, histone deacetylase 3; NCoR, nuclear receptor corepressor; SMRT, silencing mediator for retinoid and thyroid hormone receptors; DAD, deacetylase activation domain; IP_4_, inositol 1,4,5,6-tetrakisphosphate; IκBα, inhibitor of nuclear factor kappa B alpha; TNF-α, tumor necrosis factor alpha; Src, Src family tyrosine kinase; Ac, acetylation. Arrows indicate the direction of the depicted molecular transitions, trafficking events, or regulatory relationships. Colors are used to distinguish different molecular components and cellular compartments and do not indicate evidence strength. Panel (**A**) shows NCoR/SMRT-DAD–IP_4_-mediated licensing of HDAC3 catalytic activity, whereas panel (**B**) summarizes representative mechanisms of cytoplasmic anchoring, membrane association, and nuclear translocation. The TNF-α/IκBα pathway is shown as a context-specific example rather than a universal trafficking mechanism. Created with Figdraw (ID: IUUII4f415).

These spatial dynamics are particularly important in the context of immunometabolism. Inflammatory stimulation, metabolic pressure, and changes in lactate load may simultaneously alter the complex state, subcellular localization, and substrate environment of HDAC3. When HDAC3 is mainly nuclear, its action field is more closely linked to chromatin, nuclear receptors, and nuclear transcription factors. When it enters cytoplasmic or membrane-associated regions, its potential substrates may expand to signaling proteins, metabolic enzymes, or other non-histone acylated proteins. At the same time, caution is needed: the current evidence for extra-nuclear HDAC3 function remains less robust than that for its nuclear role. Extra-nuclear localization, membrane-associated distribution, and deacetylase-independent chaperone function have been experimentally supported, but whether HDAC3 carries out systematic non-histone delactylation in the cytoplasm still requires more direct validation. Figure 2 details the molecular mechanisms by which NCoR/SMRT-DAD and IP_4_ license HDAC3 catalytic activity and illustrates representative pathways that regulate its subcellular redistribution.

### 2.4. Functional Modes: Parallel Outputs of Catalytic and Non-Catalytic Actions

The structural feature of assembly dependence means that the functional output of HDAC3 follows a dual-track mode. One track is catalytic and depends on the active site, whereas the other is non-catalytic and centers on complex assembly and physical occupancy. This framework helps explain why enzymatic inhibitors do not always reproduce the phenotype of HDAC3 genetic deletion [25], suggesting that catalytic inhibition and disruption of the structural node are not biologically equivalent. Precisely because of this dual-track architecture, defining HDAC3 simply as a “deacetylase” underestimates its mechanistic complexity.

The catalytic track is the most classical form of HDAC3 function. As noted above, HDAC3 forms an activated complex with NCoR/SMRT-DAD and IP_4_ to execute deacetylation reactions on chromatin, thereby modulating local chromatin accessibility and transcriptional thresholds [7,8,20]. At this level, HDAC3 can be viewed as the catalytic core of a corepressor complex, with its main role being to alter the permissiveness and persistence of transcriptional programs by removing acyl modifications from histones and some non-histone proteins. Importantly, as the immunometabolism paradigm has evolved, class I HDACs, including HDAC1–3, have been shown in vitro to possess delactylase activity [6]. This means that the output of the catalytic track is not fixed, and that its substrate specificity may shift in response to changes in local metabolite concentrations and enzymatic parameters.

However, catalysis alone cannot fully explain HDAC3 function. Studies of hepatic metabolic regulation suggest that disruption of the integrity of the NCoR-HDAC3 complex causes severe metabolic disturbances, whereas simple inhibition of HDAC3 enzymatic activity does not produce the same effect, indicating that physical interaction itself carries key transcriptional regulatory functions [11]. You et al. further generated a DAD point-mutant mouse model in which enzymatic activity was lost but the complex remained intact, and showed that part of the biological essentiality of HDAC3 cannot be explained solely by its deacetylase activity, providing stronger support for the independence of its non-catalytic function [26]. These findings indicate that HDAC3 is not only a catalytic effector, but also a structural node within regulatory complexes.

The non-catalytic track is also reflected in the spatial transport and molecular chaperone functions of HDAC3. HDAC3 can assist phosphorylated TR2 in entering promyelocytic leukemia (PML)-associated nuclear domains, and this function does not depend on its deacetylase activity [24]. This observation suggests that in some contexts HDAC3 functions more like a protein interaction platform or trafficking regulator than a conventional enzyme. Structural biology studies further reveal that conformation and catalysis can be separable. Certain point mutations, such as R265P, allow HDAC3 to maintain a holoenzyme-like stable conformation even in the absence of activating factors, despite a complete loss of catalytic activity [27]. This further underscores the need to distinguish conformational effects from enzymatic effects when interpreting HDAC3 function.

This parallel arrangement of catalytic and non-catalytic modes has direct implications for the mechanistic inferences developed later in this review. If a given phenotype is mainly driven by HDAC3 catalytic activity, inhibition of the active pocket may be sufficient to alter that process. If, however, the phenotype depends more on complex assembly, chromatin anchoring, or spatial trafficking, simple enzymatic inhibition may fail to reproduce the consequences of genetic deletion or complex disruption. This distinction is especially important for lactylation-related questions. Whether HDAC3 changes Kla levels is a matter of the catalytic track; whether HDAC3 alters the Kla landscape through changes in complex state, localization, or substrate accessibility involves the non-catalytic and spatial regulatory tracks.

HDAC3 is therefore better defined as an immunometabolic regulatory node with dual-track output capacity. Its catalytic core determines which acyl modifications it can process, complex assembly determines when it can be activated, spatial localization determines which substrates it can access, and non-catalytic interactions determine whether it can influence transcriptional or signaling networks independently of enzymatic activity. This framework better explains the multidirectional functions of HDAC3 under homeostasis, inflammatory activation, and metabolic stress, and also provides a structural basis for distinguishing among three classes of intervention strategies: enzymatic inhibition, complex disruption, and spatial relocalization.

## 3. Classical Nuclear Functions: Transcriptional Threshold Control and Remodeling of Inflammatory Programs

**Figure 3:**
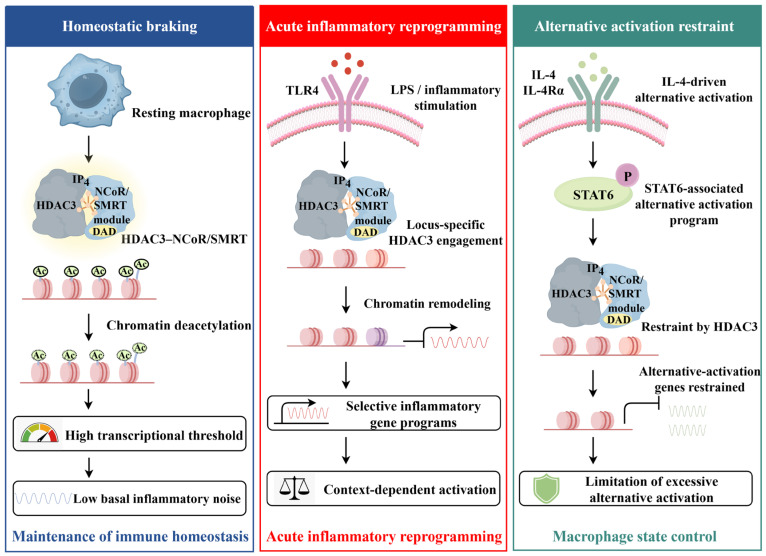
Context-dependent regulation of inflammatory transcriptional programs by HDAC3. Note: HDAC3, histone deacetylase 3; NCoR, nuclear receptor corepressor; SMRT, silencing mediator for retinoid and thyroid hormone receptors; DAD, deacetylase activation domain; IP_4_, inositol 1,4,5,6-tetrakisphosphate; LPS, lipopolysaccharide; TLR4, Toll-like receptor 4; IL-4, interleukin-4; IL-4Rα, interleukin-4 receptor alpha; STAT6, signal transducer and activator of transcription 6; Ac, acetylation; P, phosphorylation. Arrows indicate the direction of the depicted signaling, chromatin-regulatory, or transcriptional processes. Colors are used to distinguish the three functional modules and do not indicate evidence strength. This figure summarizes the context-dependent roles of HDAC3 in homeostatic braking, acute inflammatory reprogramming, and restraint of excessive IL-4-driven alternative activation. The HDAC3-associated complex in panel 2 schematically represents locus-specific chromatin engagement during acute inflammation, rather than a uniform requirement for the same NCoR/SMRT-DAD–IP_4_ complex at all LPS-responsive loci. Created with Figdraw (ID: YRYOI840fb).

HDAC3 exerts context-dependent nuclear functions rather than acting solely as a transcriptional repressor. Under homeostatic conditions, it limits basal inflammatory gene expression, whereas during acute inflammation or metabolic stress, it can support selected inflammatory programs while restraining their amplitude. This gatekeeper–instigator duality is shaped by signaling context and complex state. Figure 3 summarizes the context-dependent roles of HDAC3 in homeostatic braking, acute inflammatory reprogramming, and restraint of IL-4-driven alternative activation.

### 3.1. Homeostatic Braking: Corepressor Complexes and the Setting of Inflammatory Gene Thresholds

Resting immune cells are not transcriptionally silent. Rather, they must simultaneously satisfy two seemingly contradictory requirements. On the one hand, the basal expression of inflammatory genes must be tightly constrained to avoid low-level inflammatory noise in the absence of stimulation. On the other hand, key inflammatory genes must retain the potential for rapid activation so that they can respond promptly when pathogens or damage signals appear. The NCoR/SMRT-HDAC3 corepressor complex provides an executable mechanism for achieving both goals. This complex is selectively recruited to promoters and enhancers, where it lowers chromatin accessibility through deacetylation and thereby raises the threshold for transcriptional initiation [28]. Homeostatic braking therefore does not permanently lock genes down. Rather, it converts derepression into a rate-limiting step that can be overcome only under defined conditions.

This homeostatic brake is not equivalent to permanent silencing. A more accurate view is that HDAC3-associated corepressor complexes set an adjustable activation threshold for immune genes. Only when the strength of upstream stimulation, transcription factor occupancy, and the clearance of corepressor complexes jointly reach a certain level can these genes shift from a repressed state into an activatable one. Classical biochemical studies established that HDAC3 can be stably assembled within SMRT/NCoR complexes together with proteins such as transducin beta-like protein 1 (TBL1) and TBL1-related protein 1 (TBLR1) [29,30]. Later studies further showed that complex subunits such as G protein pathway suppressor 2 (GPS2) also participate in regulating the signaling output and stability of this system, indicating that the NCoR/SMRT-HDAC3 complex is not simply a loose protein aggregate, but a multicomponent regulatory platform capable of integrating signal input with transcriptional repression [31].

Studies in macrophages have further pushed this complex function to the level of a transcriptional checkpoint. NCoR restricts the aberrant activation of part of the activator protein 1 (AP-1)-dependent gene network, thereby preventing macrophages from prematurely entering an inflammatory program under basal conditions [32]. This model emphasizes that many inflammatory genes are not inactive in the resting state because activators are absent, but because they remain in a state that is poised for activation yet still repressed. The critical question then becomes when the corepressor complex will be removed. In the context of Toll-like receptor (TLR) signaling, derepression is neither a simple nor a uniform process, but instead displays receptor and gene selectivity. TLR2 and TLR4 can act through distinct signaling pathways at the NCoR derepression checkpoint, thereby determining whether different gene sets can cross the transcriptional threshold [33]. This mechanism makes it difficult for noisy signals to cross the gate, while ensuring that cells can release the brake rapidly when they encounter a genuine threat.

At the level of molecular execution, TBL1 and TBLR1 are regarded as important hubs for the transition from repression to activation. Phosphorylation of TBL1/TBLR1 at regulated promoter regions helps cells overcome multiple transcriptional repression checkpoints, thereby driving genes from a corepressed state into an activated one [34]. Thus, homeostatic braking depends not only on the persistent presence of HDAC3, but also on stimulus-triggered complex clearance, which directly determines whether the threshold is lowered and to what extent. In other words, HDAC3-associated complexes both suppress basal noise and preserve the structural interface required for rapid derepression later on.

Anti-inflammatory and metabolically sensing signals can also actively raise this threshold. SUMOylation of peroxisome proliferator-activated receptor gamma (PPARγ) promotes the retention of NCoR-associated complexes at inflammatory gene promoters, thereby blocking the corepressor clearance that would otherwise occur and achieving transrepression of inflammatory genes [35]. Subsequent work further suggested that liver X receptor (LXR) and PPARγ engage parallel SUMOylation-dependent transrepression pathways [36], and that cooperation between NCoR and SMRT can integrate pro-inflammatory and anti-inflammatory signals into the same corepressor-based strategy [37]. Thus, the HDAC3-associated homeostatic brake is not only an epigenetic repression mechanism, but also a key effector platform through which metabolically sensing signals regulate inflammatory thresholds.

Under repeated stimulation or chronic inflammatory conditions, threshold setting may be further transformed into selective tolerance. Nuclear factor kappa B (NF-κB)-binding motifs can specify TLR-induced gene repression programs and promote the assembly of inducible repressosome complexes at selected promoters, driving part of the inflammatory gene set into a more stable silent state [38]. This means that the HDAC3-associated corepressor system is involved not only in maintaining low noise in resting cells, but may also promote upward resetting of inflammatory thresholds during chronic stimulation or tolerance formation. In this sense, the homeostatic brake of HDAC3 does not simply block inflammation, but defines the response boundary of immune cells to external stimuli by modulating gene accessibility and the ease of derepression.

### 3.2. Acute Reprogramming: Chromatin Licensing and Gene Activation Under Inflammatory Stimulation

When inflammatory stimulation arrives, immune cells do not simply switch on all previously silent genes. Acute inflammation is better understood as a rapid and hierarchical process of chromatin reprogramming: lineage-determining factors pre-establish a responsive enhancer framework, stimulatory signals then reshape enhancer activity and transcriptional elongation, and transcription factors, coregulators, and chromatin remodeling complexes together determine the timing and intensity of gene activation. In this process, HDAC3 is not completely excluded from inflammatory programs. Instead, it is redeployed to specific regulatory regions, where it participates in gene licensing, signal amplification, and response restraint.

The inflammatory response of macrophages has a clear lineage basis. During differentiation, lineage-determining factors pre-mark a large number of potential regulatory elements, endowing these regions with the capacity to receive later stimulatory signals. This framework constrains where TLR signaling can engage and helps explain why the same stimulus elicits different gene-selective outputs in different immune cell types [39]. Tissue environments can further select and reinforce particular enhancer combinations, enabling tissue-resident macrophages to develop more environmentally specialized regulatory architectures [40]. Thus, the fact that the same inflammatory stimulus generates different outputs in different cellular or tissue contexts is not incidental, but is jointly determined by a preconfigured enhancer repertoire and local chromatin structure.

The activation of stimulus-induced latent enhancers is a defining feature of acute inflammatory reprogramming. Many enhancers lack canonical activation marks in the resting state, yet can rapidly acquire enhancer-associated histone modifications upon stimulation and enter a transcriptionally competent state [41]. Later studies further suggested that enhancer landscape rewriting is frequently coupled to enhancer transcription (eRNA production), implying that enhancer activation is not a single modification event, but a coordinated process involving chromatin opening, transcription factor binding, and local transcriptional activity [42]. This mechanism allows inflammatory cells to rapidly open new enhancer routes on demand, expanding the pool of usable regulatory elements and thereby generating highly selective gene-expression responses rather than merely accelerating pre-existing programs.

Control of transcriptional elongation at the promoter level also determines the magnitude of inflammatory gene output. Some inducible genes are already in a poised or paused state before stimulation, with transcriptional machinery preloaded; stimulatory signals can then trigger rapid high-level expression by releasing the elongation block [43]. The induction of inflammatory genes is further shaped by promoter architecture, chromatin state, and transcription factor combinations, causing different genes to exhibit early, delayed, or sustained expression kinetics [44]. At the level of chromatin remodeling, remodeling complexes such as SWI/SNF and Mi-2β play selective and sometimes antagonistic roles in inflammatory gene activation, with some genes depending more heavily on remodeling-complex engagement to cross structural barriers [45]. Acute inflammatory responses therefore exhibit a pattern of layered licensing rather than uniform global opening.

HDAC3 has context-dependent roles during acute inflammation. Traditionally, HDACs have been viewed mainly as transcriptional repressors, which can lead to underestimation of the positive contribution of HDAC3 to inflammatory activation. Genetic evidence shows that loss of HDAC3 markedly impairs the lipopolysaccharide (LPS)-induced inflammatory gene-expression program, indicating that HDAC3 is not only involved in suppressing inflammation, but also participates in the effective establishment of inflammatory transcriptional networks [9]. More specifically, the dichotomous engagement model provides a clearer explanation for the dual role of HDAC3 as gatekeeper and instigator: at some loci, HDAC3 can support inflammatory gene expression independently of NCoR1/2, whereas at other loci it uses its deacetylase activity to restrain response intensity, thereby generating a combined output of amplification and limitation in parallel [46].

HDAC3 in acute inflammation is therefore not a passive repressor withdrawn from chromatin, but a regulatory module actively involved in chromatin licensing and inflammatory output shaping. It is selectively redeployed across different gene modules and simultaneously undertakes the dual tasks of amplification and restraint. This bidirectional role makes HDAC3 more akin to a structural tuner of transcriptional programs: its function is not simply to switch inflammation off or on, but to regulate activation thresholds, output amplitude, and response duration across different gene modules.

### 3.3. Non-Histone Nodes: Deacetylation Circuits Involving Transcription Factors and Signaling Proteins

The nuclear functions of HDAC3 are not limited to histones and chromatin structure. Many transcription factors and signaling proteins are themselves regulated by lysine acetylation, and these modifications can influence DNA-binding capacity, nuclear residence time, protein–protein interactions, transcriptional activity, and the pace of feedback shutdown. By modulating such non-histone nodes, HDAC3 couples upstream inflammatory signaling to downstream transcriptional output in a tunable closed loop. This mechanistic layer further explains why HDAC3 can participate both in initiating inflammatory programs and in terminating responses or limiting signal amplitude.

One of the most representative non-histone targets of HDAC3 is NF-κB p65/RelA. Acetylation of p65 can alter its DNA-binding ability and its interaction with IκBα, thereby affecting the duration of NF-κB-dependent transcription. HDAC3 can interact with p65 and remove its acetylation marks, a process that promotes re-association of p65 with IκBα and drives NF-κB nuclear export and transcriptional termination [47]. In this context, HDAC3 acts as a shutdown factor in inflammatory responses, preventing excessive persistence of NF-κB signaling.

However, non-histone deacetylation is not always equivalent to repression. Different acetylation sites can have distinct functional consequences, and deacetylation of specific sites by HDAC3 may instead favor transcriptional activation. In the context of interleukin-1 (IL-1) signaling, HDAC3 can act as a coactivator by removing specific inhibitory acetylation marks on p65, such as lysine 122 and lysine 123 (K122/123), thereby promoting the expression of a subset of NF-κB target genes [48]. This indicates that the same category of deacetylation can produce opposite outcomes depending on the site involved, the stimulus, and the surrounding transcriptional complex. The bidirectionality of HDAC3 is thus evident not only at the chromatin level, but also at the level of transcription factor modification.

The STAT family provides another important class of non-histone regulatory nodes. Signal transducer and activator of transcription 1 (STAT1) is controlled by a critical phosphorylation-acetylation switch. STAT1 signaling is regulated by reciprocal interplay between phosphorylation and acetylation, and acetylation can influence interferon (IFN)-induced STAT1 phosphorylation, nuclear translocation, and signaling duration, while HDAC3 participates in reversing this modification switch [49]. This phospho-acetylation interplay enables HDAC3 to influence both the temporal window and the strength of IFN signaling. Signal transducer and activator of transcription 3 (STAT3) is also regulated by lysine acetylation, and acetylation of STAT3 at lysine 685 (Lys685) is essential for stable dimerization and transcriptional activation [50]. HDAC3 can form regulatory complexes with STAT3 and modulate its acetylation state, thereby participating in the control of cytokine-related transcriptional programs [51].

These non-histone regulatory circuits expand the nuclear role of HDAC3 beyond the classical model of histone deacetylation leading to chromatin compaction. Rapid-response factors such as NF-κB and STAT determine the immediate amplification and termination of inflammatory signaling, whereas nuclear receptors such as PPARγ and LXR influence slower transrepression programs through the stability of corepressor complexes [35,36]. HDAC3 lies at the intersection of these pathways. On the one hand, it participates in nuclear receptor-associated transrepression through the NCoR/SMRT complex. On the other hand, it modulates signal phase directly by deacetylating transcription factors themselves. This links acetylation-based tuning of signaling proteins with corepressor stability gating, making HDAC3 function more like a multilayered threshold regulator than a single-purpose enzyme.

Taken together, HDAC3 regulates inflammatory programs in the nucleus through at least three mechanisms. The first is chromatin-level threshold control, which mainly depends on the NCoR/SMRT corepressor complex and local histone deacetylation. The second is chromatin licensing and enhancer remodeling under acute stimulation, which determines whether inflammatory genes can be rapidly activated. The third is the non-histone deacetylation circuitry involving transcription factors and signaling proteins, which determines the timing of signal amplification, persistence, and termination. Together, these three layers explain why HDAC3 can both suppress inflammatory noise under homeostatic conditions and support selected inflammatory programs during acute stimulation, while also providing a continuous logic for the later discussion of acyl-substrate selection and functional shifting of HDAC3 under metabolic stress.

## 4. Metabolic Turning Point: HDAC3 in Immunometabolism

The initiation of an immune response is not only a cascade of signaling events, but also a reorganization of cellular energy flow and material flux. As glycolytic flux increases, the intracellular abundance of metabolites changes dramatically, directly reshaping the chemical environment in which deacylation reactions occur. For HDAC3, whose activity is highly dependent on complex state and substrate accessibility, metabolic change is not a background feature, but a key variable that determines functional output. Understanding the context-dependent output of HDAC3 therefore requires placing it within the dynamic coordinates of immunometabolism.

### 4.1. Metabolic Backdrop: Inflammation-Driven Metabolic Reprogramming and Lactate Load

Pro-inflammatory stimulation drives immune cells into a reproducible state of metabolic reprogramming. Classically activated macrophages and dendritic cells often shift from a state dominated by oxidative phosphorylation to one with elevated glycolytic flux. This Warburg-like metabolic transition not only meets the rapid demands of inflammatory responses for adenosine triphosphate (ATP), nucleotides, lipids, and reducing equivalents, but also creates new metabolic interfaces for inflammatory signaling [52]. Enhanced glycolysis is therefore not merely a byproduct of immune activation, but an important driving force across multiple phases of inflammation, including cell migration, cytokine production, inflammatory amplification, and effector execution [53].

Inflammatory signaling is tightly coupled to metabolic networks. LPS stimulation can rewire tricarboxylic acid cycle flux, leading to intracellular accumulation of intermediates such as succinate. These metabolites can in turn act as inflammatory signals; succinate, for example, stabilizes hypoxia-inducible factor 1 alpha (HIF-1α) and thereby enhances interleukin-1 beta (IL-1β) expression [54]. This indicates that metabolites can themselves function as amplifiers of inflammatory signaling. Key glycolytic enzymes can also enter inflammatory transcriptional circuits directly. Pyruvate kinase M2 (PKM2), for instance, can regulate HIF-1α activity and influence IL-1β induction, thereby coupling glycolytic flux to pro-inflammatory gene expression [55]. Together, these findings show that metabolic reprogramming in inflammatory states is not simply an alternative way to generate energy, but a regulatory system capable of feeding back onto transcriptional programs.

One direct consequence of enhanced glycolysis is an increased lactate burden. As glycolytic flux rises, glucose is continuously converted into pyruvate, accompanied by the generation of nicotinamide adenine dinucleotide (NADH). Through the lactate dehydrogenase reaction, cells reduce pyruvate to lactate and reoxidize NADH to nicotinamide adenine dinucleotide (NAD^+^), thereby sustaining the NAD^+^ demand of glycolysis [56]. This creates two immediate consequences: first, the rate of lactate production increases; second, cells face a greater acid load and a higher demand for redox turnover. In other words, the problem in inflammatory states is not simply that lactate levels rise, but that cells must continuously manage lactate, proton load, and redox balance under sustained high-flux conditions.

The monocarboxylate transporter (MCT) family provides the transmembrane outlet required for high-throughput lactate transport. MCT proteins are proton-linked monocarboxylate transporters that mediate the coupled movement of lactate and H+, thereby participating not only in metabolite export but also in pH buffering and maintenance of metabolic flux [57]. Under highly glycolytic or hypoxia-related conditions, MCT4 is particularly well suited for high-capacity lactate export, and can be upregulated through an HIF-1α-dependent mechanism, enabling cells to clear excess lactate-associated acid load more efficiently [58]. Studies have shown that MCT4 is markedly induced in inflammatory settings, where it promotes lactate export to sustain intracellular high glycolytic rates, whereas interference with MCT4 leads to abnormal lactate accumulation and reduced production of inflammatory mediators [59]. Thus, lactate export does not mean that cells have escaped metabolic pressure; rather, it is a necessary condition for maintaining high glycolytic throughput. Persistent high production and high export together create a pronounced state of lactate overload both inside and outside the cell.

At the tissue level, sustained lactate generation and export produce local lactate exposure within inflammatory lesions, tumor microenvironments, and sites of chronic inflammation. The accumulation and movement of lactate across intracellular and extracellular compartments allow it not only to reflect glycolytic intensity, but also to act as a microenvironmental signal that shapes the behavior of neighboring immune cells [60,61]. This means that HDAC3 operates against a lactate-load field jointly determined by glycolytic flux, transporter burden, and the local microenvironment. This metabolic backdrop provides the foundation for understanding how lactate may influence deacylation systems and the writing of Kla.

### 4.2. Metabolite Effects: Temporal Regulation of Deacylation Systems by Lactate

In the immune system, lactate has the dual identity of a metabolic substrate and a signaling molecule. It can participate in carbon reutilization and energy conversion, while also acting as a microenvironmental signal that shapes immune-cell phenotypes. The influence of lactate on immune phenotype and transcriptional programs is context dependent. In chronic inflammatory environments, local lactate accumulation can drive metabolic rewiring in CD4^+^ T cells and promote the maintenance of disease-associated phenotypes [61], suggesting that lactate is not only a product of inflammatory metabolism, but may also help sustain the inflammatory ecosystem itself. Lactate is therefore better understood as a regulatory variable that changes over time and space, rather than as a simple metabolic byproduct.

Lactate can also influence immune and metabolic signaling through membrane receptor pathways. G protein-coupled receptor 81/hydroxycarboxylic acid receptor 1 (GPR81/HCAR1) was initially identified as an endogenous receptor for lactate and shown to mediate the inhibitory effect of lactate on lipolysis [62]. Subsequent studies further expanded the role of HCAR1 signaling to include inflammatory regulation, metabolic adaptation, and remodeling of the tumor microenvironment [60]. This body of evidence indicates that lactate does not act only by entering intracellular metabolic networks, but can also alter cellular responsiveness to external stimuli through receptor-mediated signaling. For HDAC3, this means that lactate may reshape the deacylation environment not only through a metabolite-to-epigenetic route, but also indirectly by modulating upstream inflammatory transcriptional networks through membrane receptor signaling.

At the epigenetic level, lactate may directly alter HDAC-associated deacylation flux. Free lactate at physiological and pathological concentrations can function as an endogenous pan-HDAC inhibitor and promote histone hyperacetylation together with changes in gene expression [4]. Mechanistically, this may create a potential inhibitory window: when lactate burden becomes sufficiently high and the biochemical conditions are permissive, deacetylation flux may decline while local acetylation levels rise, thereby lowering the activation threshold of certain gene modules or altering enhancer activity, which may favor rapid opening of enhancers and promoters during early inflammation. More importantly, this metabolic inhibitory effect may be uncoupled from protein recruitment. In other words, even if HDAC3 remains bound to specific chromatin loci, its catalytic output may still be rendered inefficient by metabolic inhibition. The existence of such a window offers one possible explanation for the temporal logic of rapid opening during early inflammation followed by later reconvergence, although any such formulation must remain cautious and sensitive to experimental context.

The effect of lactate on the acetylation system does not arise only from the erasing side. The writing of histone acetylation depends on acetyl coenzyme A (Acetyl-CoA), and the pathways that generate Acetyl-CoA directly connect metabolic state to histone acetylation levels [63]. Recent work further shows that lactate can enter nuclear carbon flux through oxidative metabolism and become a major carbon source for histone acetylation, thereby enhancing substrate supply to the acetylation-writing arm [64]. This means that lactate may affect both the writing and erasing sides across different temporal windows. On the one hand, lactate burden may suppress HDAC-related deacylation capacity; on the other hand, lactate-derived carbon flux may strengthen acetylation writing by increasing Acetyl-CoA supply.

The relationship between lactate and HDAC3 therefore cannot be reduced to the statement that “lactate inhibits HDAC3.” A more appropriate interpretation is that lactate changes the metabolic coordinates within which HDAC3 operates. The supply of acetylation substrates, the efficiency of deacetylation reactions, the burden of Kla writing, and the relative proportions of multiple acyl modifications may all shift over time. As a complex-dependent deacylase, the functional output of HDAC3 is therefore more likely to depend on the combination of these variables than on lactate concentration alone. In this sense, lactate provides the metabolic basis for the rheostat model of HDAC3: the same HDAC3 complex may face entirely different modification spectra and substrate priorities under different degrees of lactate pressure.

### 4.3. Paradigm Shift: Lysine Lactylation and Metabolic-Epigenetic Coupling

The emergence of lysine lactylation has moved lactate research from the idea that a metabolite influences epigenetic enzyme activity to the broader concept that a metabolite can itself be written into protein modification networks. Enhanced glycolysis and lactate accumulation promote histone Kla and are linked to changes in the expression of specific gene sets, thereby establishing a more direct molecular route connecting metabolic flux, chromatin modification, and transcriptional programs [5]. Within this framework, lactate is no longer just an indirect indicator of metabolic state, but becomes a regulatory signal that can be read by the nucleus and by protein networks.

A key feature of the Kla paradigm is reversibility. Systematic biochemical evidence has shown that class I HDACs, especially HDAC1–3, possess delactylase activity [6]. This places HDAC3 at the center of the immunometabolic turning point. HDAC3 may not only be influenced by the effect of lactate burden on HDAC activity, but may also directly participate in erasing Kla marks. The question therefore shifts from whether lactate affects HDAC activity to how HDAC3 allocates its catalytic capacity among acetylated, lactylated, and other acylated substrates in the setting of enhanced glycolysis and increased Kla writing.

At the same time, the writing mechanism of Kla has also been substantially clarified. Recent studies have shown that histone acetyltransferase binding to ORC1 (HBO1) can function as a key lysine lactyltransferase, displaying catalytic preference for sites such as histone H3 lysine 9 lactylation (H3K9la) in cells [65]. This finding suggests that Kla is not merely a passive chemical consequence of elevated lactate levels, but may instead be regulated by a more specialized enzymatic writing system. Even more importantly, the substrate range of Kla is expanding from histones to non-histone networks. Proteomic studies have identified large numbers of non-histone lactylation sites in contexts related to neural excitation, tumors, and multiple diseases, indicating that the lactylome can encompass metabolic enzymes, nuclear regulatory factors, signaling proteins, and inflammatory effector molecules [66,67,68].

More critically, non-histone lactylation has already been shown to have clear functional consequences. In immune-related settings, lactate can promote lactylation of high mobility group box 1 (HMGB1) in macrophages and influence its extracellular release, thereby linking this modification to inflammatory amplification during sepsis [69]. In the tumor microenvironment, lactylation-driven methyltransferase-like 3 (METTL3)-m6A signaling can promote the immunosuppressive phenotype of myeloid cells [70]. The key metabolic enzyme PKM2 has also been reported to undergo lactylation, which is associated with metabolic adaptation and phenotypic shifts in pro-inflammatory macrophages [71]. Together, these findings show that lactylation is no longer merely a new chromatin mark, but a regulatory layer that can enter core processes such as immunosuppression, inflammatory amplification, and metabolic remodeling.

At the same time, the chemical space of lactate-related lysine modifications continues to expand. Glycolytic byproducts can drive lysine lactoylation through non-enzymatic pathways, especially on highly exposed proteins such as glycolytic enzymes [72]. This means that the field must carefully distinguish among enzymatic Kla, non-enzymatic lactoylation, and different forms of lactate-related modification. This distinction is particularly important for HDAC3, because the substrate environment it faces is not a single uniform pool of Kla, but rather a mixed substrate field shaped jointly by metabolic flux, acyl donors, enzymatic writing, non-enzymatic modification, and spatial distribution.

The essence of this metabolic turning point is therefore a shift in the mode of information encoding. Enhanced glycolysis does not simply alter energy supply; it also changes the dynamic balance among acyl modifications such as acetylation and lactylation. Lactate does not merely affect HDAC activity; it may also function as an information carrier within protein modification networks. HDAC3 occupies a key position within this transition, processing different acyl modifications in an environment jointly constrained by complex assembly, substrate accessibility, and metabolic pressure. This framework lays the foundation for the next section, which will discuss the kinetic interplay between acetylation and lactylation, the redistribution of catalytic resources, and the boundaries of current evidence regarding HDAC3.

## 5. Catalytic Redistribution: Kinetic Interplay and the Boundaries of Current Evidence 

**Figure 4:**
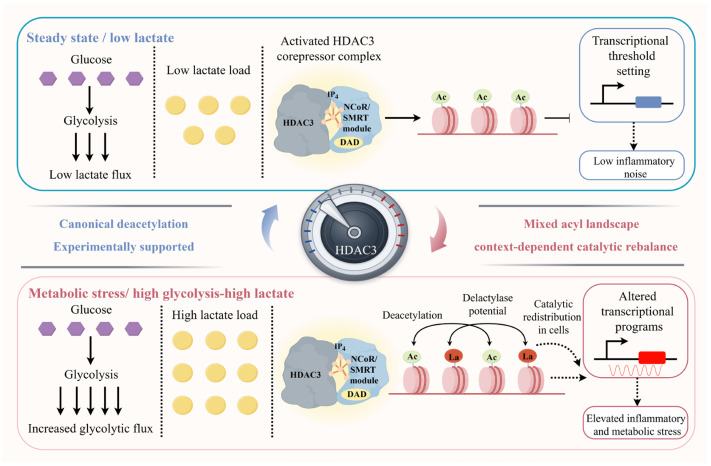
Metabolic–epigenetic balance and context-dependent redistribution of HDAC3 catalytic output. Note: HDAC3, histone deacetylase 3; NCoR, nuclear receptor corepressor; SMRT, silencing mediator for retinoid and thyroid hormone receptors; DAD, deacetylase activation domain; IP_4_, inositol 1,4,5,6-tetrakisphosphate; Ac, acetylation; La, lactylation. Solid arrows indicate experimentally supported activities, including canonical deacetylation and biochemically demonstrated delactylase activity. Dashed arrows indicate model-supported cellular redistribution that requires further validation. Colors are used to distinguish different metabolic states and functional modules and do not indicate evidence strength. The upper panel shows a deacetylation-dominant state under low-lactate conditions, whereas the lower panel presents a proposed context-dependent redistribution of HDAC3 catalytic output across multiple acyl substrates under conditions of high glycolytic flux and lactate load. Created with Figdraw (ID: YTYWY5a1a1).

HDAC3 is increasingly recognized as a deacylation node capable of processing multiple acyl modifications rather than solely as a deacetylase. During inflammation and metabolic reprogramming, its catalytic output is jointly shaped by metabolite supply, complex assembly, substrate accessibility, and enzyme kinetics. Figure 4 contrasts the experimentally supported deacetylation-dominant state under low-lactate conditions with a proposed context-dependent redistribution across acyl substrates under high glycolytic flux and lactate load.

### 5.1. Catalytic Potential: The Biochemical Basis for HDAC3 as a Lysine Delactylase

The HDAC family was originally defined by deacetylase activity, yet the substrate boundaries of class I HDACs are no longer confined to acetyl groups. Crotonylation provides a representative example. Class I HDACs have been identified as major histone decrotonylases, and HDAC1 and HDAC3 mutants can exhibit partial uncoupling between deacetylase and decrotonylase activities [15,17]. This indicates that the catalytic system of class I HDACs is intrinsically capable of processing larger short-chain acyl groups, thereby providing a reasonable chemical basis for HDAC3 to act on lactyl-lysine.

From a structural perspective, the active pocket and hydrophobic tunnel of HDAC3 provide the spatial basis for accommodating short-chain acyl substrates. The catalytic pocket and hydrophobic tunnel of class I HDACs display considerable extensibility and conformational plasticity, allowing them to accommodate acyl side chains bulkier than acetyl groups [73]. This point is particularly important for HDAC3, because its efficient catalysis does not primarily depend on the free-enzyme state, but on the activated conformation formed through NCoR/SMRT-DAD and IP_4_. Whether HDAC3 can process a given acyl substrate therefore cannot be explained solely by chemical compatibility at the active pocket, but must also take into account complex state and substrate-delivery context.

The key evidence directly supporting the delactylase capacity of HDAC3 comes from systematic enzymatic profiling. Moreno-Yruela et al. compared HDAC and sirtuin family members and found that class I HDAC1–3 are the most effective lysine delactylases in vitro, with HDAC1 and HDAC3 also showing a degree of site-specific delactylase activity in cells [6]. Zessin et al. subsequently examined acyl specificity more broadly across zinc-dependent HDACs and sirtuins, further supporting robust delactoylase activity for HDAC3 [74]. Together, these two lines of evidence show that HDAC3 is not only theoretically capable of recognizing lactyl-lysine, but already has clear in vitro enzymatic support as a delactylase.

Quantitative kinetic data further define the biochemical delactylase capacity of HDAC3, although these values remain dependent on substrate sequence, complex preparation, and assay format. Using an NCoR2-DAD-activated HDAC3 complex and Ac-LGK-AMC model substrates, hydrolysis of L-lactyl-lysine exhibited a *K*_M_ of 122 ± 7 μM and a kcat of 0.49 ± 0.02 s^−1^, corresponding to a catalytic efficiency of approximately 4.0 × 10^3^ M^−1^·s^−1^. The corresponding D-lactyl-lysine substrate exhibited a *K*_M_ of 67 ± 7 μM and a *k*_cat_ of 0.92 ± 0.04 s^−1^, yielding a catalytic efficiency of approximately 1.37 × 10^4^ M^−1^·s^−1^. In the same substrate series, short linear acyl modifications, including Kac, generally displayed *K*_M_ values below 10 μM and catalytic efficiencies above 1 × 10^5^ M^−1^·s^−1^. Thus, on this model sequence, HDAC3 processes acetyl-lysine at least 25-fold more efficiently than L-lactyl-lysine. The greater efficiency toward D-lactyl-lysine primarily demonstrates stereochemical sensitivity in the model assay; its physiological relevance should be interpreted cautiously because glycolysis-responsive histone lactylation in mammalian cells is predominantly L-lactylation [6,75]. These measurements establish the intrinsic delactylase capacity of purified HDAC3 in vitro but do not, by themselves, demonstrate HDAC3-specific lactylation editing in cellular or in vivo settings.

This kinetic difference imposes an important constraint on the proposed catalytic-redistribution model. The available biochemical data do not support an inversion of the intrinsic catalytic preference of HDAC3 from Kac to Kla. However, catalytic efficiency measured using an isolated fluorogenic peptide does not by itself determine cellular deacylation flux. Net substrate processing in cells is also influenced by the effective local abundance and accessibility of individual modified sites, HDAC3 recruitment and complex state, substrate sequence and structural context, and subcellular compartmentalization. Acetylated and lactylated substrates therefore need not constitute a single homogeneous pool undergoing uniform competition for the same HDAC3 molecules. Increased glycolytic flux and lactate load may increase the formation or turnover demand of selected Kla sites, but whether local substrate enrichment or spatial recruitment is sufficient to offset the intrinsic kinetic preference for Kac remains unknown [6,73,76,77].

These biochemical findings should not be interpreted as establishing a fixed activity hierarchy among HDAC1–3. HDAC1–3 all display substantial delactylase activity in vitro, but their relative activities vary with substrate sequence, lactylation site, lactyl isomer, enzyme or complex preparation, and assay format. Evidence derived from these purified-enzyme systems should therefore be distinguished from cell-based perturbation studies and from findings obtained in animal models or human tissues. In the initial cell-based analyses, HDAC1 and HDAC3 produced prominent site-specific changes in histone lactylation, whereas an independent biochemical study identified robust delactylase activity for both HDAC2 and HDAC3 [6,74]. More recent context-specific studies have further identified HDAC1-mediated H4K12 delactylation, HDAC2-mediated METTL3 K27 delactylation, and HDAC3-mediated regulation of histone and non-histone lactylation, including H4K5la and RHOA K118/K162la [78,79,80]. These examples support isoform- and substrate-dependent specialization, but they do not yet provide a systematic head-to-head comparison or establish HDAC3 as the universally predominant delactylase.

Cell-based evidence for HDAC3-specific lactylation editing remains more limited and site dependent. HDAC3 overexpression produced detectable regulation of H4K5la but did not consistently alter global Kla or H3K18la, supporting site-selective rather than uniform control of the cellular histone lactylation landscape [6]. More recent work identified RHOA K118 and K162 as non-histone lactylation sites regulated by HDAC3, supported by cellular perturbation, recombinant-protein delactylation assays, and site-specific lactylation analyses [80]. Although the accompanying xenograft and human-tumor findings support the pathological relevance of RHOA lactylation, they do not independently establish direct HDAC3-catalyzed delactylation of these sites in vivo. These findings provide evidence for selected HDAC3-dependent cellular delactylation events, but they do not establish a global redistribution of HDAC3 catalytic capacity between cellular Kac and Kla pools under lactate load. Taken together, these findings establish a biochemical basis for HDAC3-mediated delactylation and provide emerging evidence for selected cellular targets, but they do not yet define its broader physiological contribution to Kla removal or support global catalytic redistribution under lactate load.

Importantly, having delactylase activity is not equivalent to being the dominant Kla eraser in all cellular contexts. The canonical deacetylase activity of HDAC3 depends strongly on the activated conformation formed by NCoR/SMRT-DAD and IP_4_ [7,8], and proper C-terminal conformational organization has also been shown to be important for DAD/IP_4_-dependent activation [81]. At the same time, in vivo studies indicate that some biological functions of HDAC3 cannot be fully explained by loss of catalytic activity alone, suggesting that complex integrity and catalytic activity can, under certain conditions, be partially separable [26]. A more cautious conclusion is therefore that the biochemical identity of HDAC3 as a delactylase is now supported by strong evidence, but whether the endogenous HDAC3-NCoR/SMRT-IP_4_ complex serves as a major Kla eraser in inflammatory cells still requires more direct cellular and in vivo validation.

This evidentiary boundary is central to the logic of the present review. The delactylase activity of HDAC3 qualifies it to enter the Kla regulatory network, but its actual functional output remains dependent on complex assembly, substrate sites, subcellular distribution, and metabolic pressure. In other words, HDAC3 should not be described simply as an enzyme that already comprehensively governs Kla erasure. Rather, it is better defined as a complex-dependent deacylation node with clear delactylase potential that may participate in Kla dynamics under specific metabolic conditions.

### 5.2. Kinetic Interplay: Acyl Preference Shaped Jointly by Substrate Supply and Enzymatic Parameters

The acyl preference displayed by HDAC3 in cells cannot be determined by the active pocket alone. In authentic cellular settings, substrate preference is better understood as an emergent property jointly shaped by substrate supply, compartmentalized donor pools, complex assembly, the local site environment, and the burden of competing modifications. The relationship between acetylation and lactylation likewise should not be reduced to two substrates competing equally for the same catalytic pocket, but must instead be interpreted within the broader context of shifting metabolic phases and remodeling of modification networks.

First, the writing of acetylation and lactylation is constrained by different metabolic donor pools. Acetylation depends on acetyl coenzyme A (Acetyl-CoA), and both the abundance and compartmentalization of Acetyl-CoA directly influence histone acetylation levels [63]. Acyl-CoA metabolism is not evenly distributed throughout the cell; rather, distinct intracellular compartments contain clearly segregated acyl-CoA pools, which affects the probability and site selectivity of different acyl modifications. In this sense, substrate preference is constrained by the spatial and quantitative properties of donor pools [77]. Studies on metabolic regulation of histone modifications likewise suggest that enzymatic parameters acquire biological meaning only when the relevant substrate supply is truly present and reaches the appropriate compartment [76]. Thus, the so-called competition between acetylation and lactylation first emerges at the level of metabolic supply and compartmental distribution, and only then propagates to the erasing side, rather than reflecting two substrates simply entering the same active pocket at the same time.

Second, lactylation itself is not a single, homogeneous modification pool. Lactoyl-CoA has been quantitatively detected in mammalian cells and tissues, but its overall abundance and dynamic range must still be evaluated in a context-dependent manner [82]. This means that the lactyl donor pool should not be assumed to overwhelm Acetyl-CoA under all inflammatory conditions. More recently, glycolysis-induced histone lactylation has been shown to occur predominantly in the form of L-lactylation, indicating that not all lactate-related lysine modifications are equivalent [75]. Studies of non-enzymatic lactoylation further show that glycolytic byproducts can modify proteins such as glycolytic enzymes through lactoylglutathione-related pathways [72]. Thus, the substrate field encountered by HDAC3 in real cells is not a pure Kla pool, but a mixed acyl environment composed of enzymatic Kla, non-enzymatic lactoylation, different isomers, and distinct subcellular compartments. Acyl preference is therefore better viewed as a net response to this mixed substrate background.

Moreover, acyl preference on the erasing side is not fixed. Different zinc-dependent HDACs vary in both deacetylase and broader deacylase capacity, indicating that HDAC isoforms differ in substrate preference and catalytic efficiency [16]. Further studies show that class I HDAC1–3 usually do not function as free enzymes in cells, but instead operate within distinct multiprotein complexes, and these complex states affect substrate recognition, nucleosomal site selectivity, and overall catalytic behavior [73]. For HDAC3, this complex effect is especially important, because its most typical high-activity state requires the licensing of NCoR/SMRT-DAD-IP_4_ assembly [7,8]. The preference of HDAC3 for acetyl or lactyl groups should therefore not be written as a static property, but rather understood as a kinetic feature jointly determined by the local metabolic environment and complex state.

Accordingly, the kinetic interplay between acetylation and lactylation is better described as a multilayered coupling among metabolic supply, writing burden, complex state, and erasing capacity. Under low-lactate homeostatic conditions, HDAC3 is more likely to function predominantly through the canonical NCoR/SMRT complex to maintain deacetylation and transcriptional threshold control. Under high glycolytic flux and high lactate load, however, increased Kla writing, expansion of the non-histone lactylome, and redistribution of HDAC3 complexes may together alter the weighting of its substrate processing. It is important to emphasize that this process still should not be directly described as competitive inhibition of the HDAC3 active center by lactylated substrates. A more careful formulation is that under high lactate load, the burden of lactylated substrates and related acyl modifications may increase the complexity of substrate processing by HDAC3 and thereby change its net catalytic output; whether this constitutes strict competitive inhibition remains to be tested by dedicated kinetic experiments.

### 5.3. Context Model: Metabolic-State-Dependent Redistribution of HDAC3 Output

Based on current evidence, changes in HDAC3 functional output across metabolic states can be conceptualized as a context-dependent redistribution model. *The proposed context-dependent redistribution model suggests that increased glycolytic flux and lactate load may alter the relative contribution of HDAC3 to canonical deacetylation and the processing of other acyl substrates, with the resulting output shaped by NCoR/SMRT-dependent complex state, metabolic and acyl-substrate availability, and compartment-specific substrate access*. These variables should be understood as interacting regulatory inputs rather than as interchangeable or independently sufficient determinants. Accordingly, the model does not assume simple or uniform competition between acetylated and lactylated substrates for the HDAC3 active site. Here, “catalytic redistribution” denotes a possible context-dependent change in the relative contribution of HDAC3 to distinct acyl-substrate pools, rather than a demonstrated inversion of its intrinsic Kac versus Kla preference or a global transition from deacetylation to delactylation. This model is summarized schematically in Figure 4. Under low-lactate or homeostatic conditions, a relatively stable balance is maintained among Acetyl-CoA supply, canonical histone acetylation, and HDAC3–NCoR/SMRT complex-mediated deacetylation. In this setting, HDAC3 functions predominantly as a deacetylation-oriented homeostatic gatekeeper, maintaining low basal expression and transcriptional thresholds of inflammatory genes through corepressor complexes. This does not imply that HDAC3 lacks delactylase capacity; rather, under this metabolic background, the contribution of Kla writing and erasing to the overall transcriptional output is likely to be comparatively limited.

This balance may shift during acute metabolic stress. Enhanced glycolysis increases lactate flux and changes both donor pools and modification spectra. At the same time, inflammatory signaling itself can alter HDAC3 localization, chromatin occupancy, and complex assembly state [7,8,11,26]. Under these conditions, HDAC3 may experience three simultaneous pressures: potential regulation of HDAC-associated activity by lactate burden, increased erasing demand due to Kla/lactylome expansion, and altered substrate recognition together with local activity caused by changes in complex state [4,6,73]. These factors do not necessarily force HDAC3 to leave chromatin; rather, they are more likely to alter its net catalytic output, shifting it from a relatively single deacetylation-dominant state into a rebalancing state in which multiple acyl substrates coexist and compete for processing. 

This model must also accommodate a newly emerging layer of complexity. Recent biochemical studies suggest that class I HDACs can catalyze lysine lactylation under appropriate donor conditions [83]. This does not overturn the conclusion that HDAC3 can remove lactyl modifications; rather, it suggests that the class I HDAC system may operate more like a reversible acyl-exchange network whose direction depends on local donor and acceptor concentrations, the chemical environment, and complex state. In other words, HDAC3 is not necessarily a one-way eraser. Under metabolic pressure, it may become an acyl-processing node with directional bias. This further reinforces the central view of HDAC3 as a rheostat rather than a single-function executor.

The redistribution model proposed here should therefore be understood as a context model grounded in relatively strong biochemical evidence, rather than as a fully established unifying mechanism. It yields three clear experimental predictions. First, the relative contribution of HDAC3 to Kac and Kla turnover at defined sites should change as metabolic pressure varies. Second, disrupting DAD/IP_4_-mediated complex assembly and simply inhibiting the catalytic pocket of HDAC3 should not produce identical changes in Kla and Kac profiles. Third, expansion of the extra-nuclear lactylome may be functionally coupled to HDAC3 spatial relocalization, complex reorganization, or non-catalytic interactions. At present, the most cautious conclusion is that HDAC3 as a delactylase is supported by relatively clear direct evidence, whereas whether it undergoes catalytic redistribution between acetylation and lactylation still awaits further quantitative validation at the cellular and in vivo levels. To distinguish direct evidence, indirect support, and model-based inference, Table 1 systematically summarizes the current evidence spectrum for HDAC3-mediated deacetylation and delactylation.

## 6. Cytoplasmic Frontier: Extra-Nuclear HDAC3

Although HDAC3 is classically viewed as a nuclear, complex-dependent regulator of chromatin and transcriptional thresholds, spatial localization studies and expanding non-histone lactylation maps suggest broader extra-nuclear functions. Under high glycolytic flux and lactate load, HDAC3 may redistribute to cytoplasmic or membrane-associated compartments, where it could access lactylated innate immune sensors, inflammasome-related proteins, and metabolic enzymes. This extra-nuclear pool may therefore contribute to signal compartmentalization and candidate cytoplasmic delactylation networks, although direct substrate-specific evidence remains limited.

### 6.1. Spatial Relocalization: Inflammatory Signaling and Complex Anchoring Jointly Shape Functional Compartmentalization

The extra-nuclear functions of HDAC3 first depend on whether it possesses a regulatable capacity for subcellular redistribution. Early domain-mapping studies showed that HDAC3 does not statically reside in the nucleus: its middle region is associated with extra-nuclear distribution, whereas its C-terminal region is more closely linked to nuclear enrichment and transcriptional repression [21]. Subsequent functional studies further indicated that the nuclear localization signal region of HDAC3 is necessary and sufficient for its nuclear functional output, and that alterations in this localization module affect its ability to regulate target-gene expression [22]. Together, these findings indicate that the nucleocytoplasmic distribution of HDAC3 is not a simple passive process, but rather a tunable one constrained by intrinsic localization modules.

This spatial plasticity becomes more evident under inflammatory conditions. Under basal conditions, HDAC3 can associate with IκBα and remain more cytoplasmically distributed. When TNF-α induces IκBα degradation, HDAC3 translocates into the nucleus and participates in TNF-α-mediated repression of PPARγ transcriptional activity [23]. The significance of this finding lies not only in showing that HDAC3 can undergo nuclear translocation, but also in indicating that partner proteins can reshape its subcellular action range by anchoring or releasing it. In this context, IκBα is not only a classical inhibitor of NF-κB signaling, but also a regulator of HDAC3 spatial distribution that links inflammatory signaling to HDAC3 functional localization.

HDAC3 also exhibits spatial redirection beyond canonical nucleocytoplasmic shuttling. It can localize to the plasma membrane and form a complex with c-Src, while itself serving as a substrate of Src [12]. In addition, HDAC3 can exert deacetylase-independent molecular chaperone functions by facilitating the transport of phosphorylated TR2 from the cytoplasm to PML-associated nuclear domains [24]. Together, these findings indicate that HDAC3 is not merely an enzyme that performs chromatin deacylation in the nucleus. Under certain conditions, it can also participate in signal compartmentalization through protein interaction, spatial trafficking, and non-catalytic scaffold-like functions.

Spatial distribution and complex state are likewise not independent of one another. Recent studies in macrophages suggest that SMRT not only functions as a transcriptional corepressor, but also serves as a chromatin anchor for the HDAC3 complex. When SMRT is depleted, chromatin binding of the complex is markedly impaired, accompanied by weakened nuclear localization of HDAC3-associated complexes [85]. This evidence moves the spatial function of HDAC3 beyond the simple idea of a single protein shuttling in and out of the nucleus and toward the broader level of complex localization and anchoring. The appearance of HDAC3 outside the nucleus should therefore not be interpreted simply as random diffusion after dissociation from its original complex, but more likely as a rebalancing among complex assembly, chromatin anchoring, and signal-induced localization.

The extra-nuclear significance of HDAC3 is thus founded first on spatial plasticity. Current evidence already supports its capacity for nucleocytoplasmic shuttling, membrane-associated localization, and non-catalytic trafficking functions. However, these findings are still insufficient to prove directly that HDAC3 carries out systematic delactylation in the cytoplasm. A more cautious formulation is that HDAC3 possesses the spatial basis for entering extra-nuclear action fields, whereas whether extra-nuclear delactylation truly occurs must still be validated in conjunction with specific substrates, specific modification sites, and functional readouts.

### 6.2. Non-Canonical Substrate Layer: The Whole-Cell Lactylome and Its Functional Consequences

Whether extra-nuclear function is mechanistically meaningful depends critically on whether an important and functionally relevant substrate background exists outside the nucleus. One of the most significant advances in lactylation research over the past few years has been the rapid expansion from histone Kla to the whole-cell protein lactylome. Studies in neural excitation models have shown that protein lactylation is not restricted to histones, but occurs widely across brain tissue [66]. Proteomic analyses of hepatocellular carcinoma samples have likewise identified large numbers of lysine lactylation sites, suggesting that the lactylome can encompass metabolic enzymes, signaling proteins, and multiple classes of regulatory factors [67]. Although these studies are not centered on HDAC3, they are already sufficient to show that lactylation is no longer simply a chromatin event, but a broadly distributed modification background extending across whole-cell protein networks.

More importantly, non-histone lactylation has already been shown to produce clear functional consequences, rather than merely existing as a static mark. Lactylation together with acetylation of HMGB1 promotes its exosomal release and has been linked to inflammatory amplification in sepsis [69]. Lactylation of PKM2 can reshape its metabolic adaptation and pro-inflammatory functional state [71]. In the tumor microenvironment, lactylation-driven METTL3-associated m6A signaling can enhance the immunosuppressive phenotype of tumor-infiltrating myeloid cells [70]. Together, these studies indicate that cytoplasmic or whole-cell lactylation is not simply an incidental byproduct of metabolic stress, but a regulatory layer that can directly participate in key processes such as inflammation, metabolic adaptation, and immune suppression.

Non-enzymatic lactate-related modifications further expand the complexity of the extra-nuclear substrate layer. Glycolytic byproducts can induce lysine lactoylation through non-enzymatic routes, particularly on highly exposed proteins such as glycolytic enzymes [72]. This suggests that cytoplasmic proteins do not face a single, homogeneous enzymatic Kla network, but rather a mixed modification environment jointly shaped by enzymatic lactylation, non-enzymatic lactoylation, metabolic flux, and protein exposure. This background is particularly important for HDAC3, because it means that potential extra-nuclear substrates are not limited to a few isolated proteins, but may instead form a dynamic protein-modification network that changes with glycolysis and lactate load.

Recent work has pushed this substrate layer even further toward innate immune sensing. L-lactate has been shown to be sensed by Alanyl-tRNA Synthetase 1 (AARS1) and Alanyl-tRNA Synthetase 2 (AARS2), which in turn drive global lysine lactylome formation and specifically regulate lactylation and inactivation of cyclic GMP-AMP synthase (cGAS) [86]. This finding shifts lactylation from being merely a consequence of elevated lactate to a protein-modification program organized by specific lactate-sensing and transfer systems. Building on this, cGAS lactylation has also been shown to promote its ubiquitin-independent proteasomal degradation, thereby weakening innate immune surveillance [88]. The importance of this body of evidence lies in showing that non-histone lactylation is no longer simply a broadly present protein modification, but has already reached a level at which it can directly reshape the fate of key signaling nodes.

The cytoplasmic and whole-cell lactylome thus already satisfies two conditions: the substrate range is broad enough, and several of its nodes have demonstrated observable functional consequences. The potential extra-nuclear significance of HDAC3 is built precisely on this realistic substrate background. However, the current evidence still needs to be carefully stratified. The existence and functional consequences of non-histone lactylation are already relatively clear, whereas whether HDAC3 directly erases these cytoplasmic lactylation sites still requires confirmation through targeted mass spectrometry, site-directed mutagenesis, complex perturbation, and layered genetic or pharmacologic dissection of HDAC3. Available HDAC1–3-dependent delactylation examples remain context specific, and isoform-resolved subcellular delactylomes have not yet been systematically compared. These reported substrates should therefore not be interpreted as evidence for fixed isoform-specific or compartment-wide substrate preferences.

### 6.3. Candidate Functional Modules: Innate Immune Sensors, Inflammasomes, and Metabolic Enzyme Networks

The extra-nuclear roles of HDAC3 are currently best considered through candidate functional modules rather than as a collection of validated cytoplasmic delactylation substrates. The first module that deserves priority attention is the axis of innate immune sensors and antiviral signaling. TANK-binding kinase 1 (TBK1) is a central kinase within this module, and HDAC3 can directly deacetylate TBK1 and enhance its kinase activity, thereby promoting IRF3-related innate antiviral responses [89]. This provides direct evidence that HDAC3 can regulate cytoplasmic immune signaling proteins, although the current support primarily concerns deacetylation rather than delactylation.

Closely linked to the TBK1 axis is the cGAS-dependent DNA-sensing pathway. AARS1/AARS2-mediated lactylation of cGAS suppresses its activity, and subsequent work further suggests that cGAS lactylation promotes its ubiquitin-independent degradation [86,88]. In parallel, HDAC3 can also cooperate with Forkhead box K1 (FOXK1) to sustain STAT1/STAT2 transcription, thereby supporting antiviral innate immune programs in macrophages [90]. Taken together, although these lines of evidence do not yet justify the direct conclusion that HDAC3 delactylates cGAS, they are sufficient to outline a clear candidate framework: the TBK1-cGAS-STAT1/2 module, spanning cytoplasmic and nucleocytoplasmic innate immune signaling, is likely to represent one of the most promising functional regions in which to track extra-nuclear deacylation by HDAC3.

A second module centers on the inflammasome and IL-1β output. HDAC3 can reconfigure mitochondrial fatty acid oxidation through non-histone deacetylation, thereby driving IL-1β-dependent inflammatory responses in macrophages [84]. Although this finding does not itself involve lactylation, it clearly demonstrates that HDAC3 can materially engage the extra-nuclear execution layer of the NLRP3/IL-1β axis. More importantly, NLRP3 itself has now been shown to undergo lactylation, and lactylation at K24 and K565 promotes inflammasome activation [87]. This moves NLRP3 beyond the status of a theoretically lactylatable candidate and into the category of a protein with direct lactylation evidence and demonstrated functional consequences.

A third module involves metabolic enzymes and the broader coupling between metabolism and inflammation. Lactylation of PKM2 has been shown to alter the metabolic adaptation of pro-inflammatory macrophages [71], while studies of non-enzymatic lactoylation further indicate that glycolytic enzymes themselves are situated in a high-exposure environment for lactate-related modifications [72]. These findings imply that if HDAC3 indeed performs cytoplasmic delactylation, its likely substrates may not be limited to classical inflammatory signaling proteins but may also include enzymatic nodes that determine local metabolic flux and inflammatory intensity. The metabolic-enzyme network therefore should not be regarded merely as a background system but may instead constitute an important platform for the extra-nuclear actions of HDAC3.

**Figure 5:**
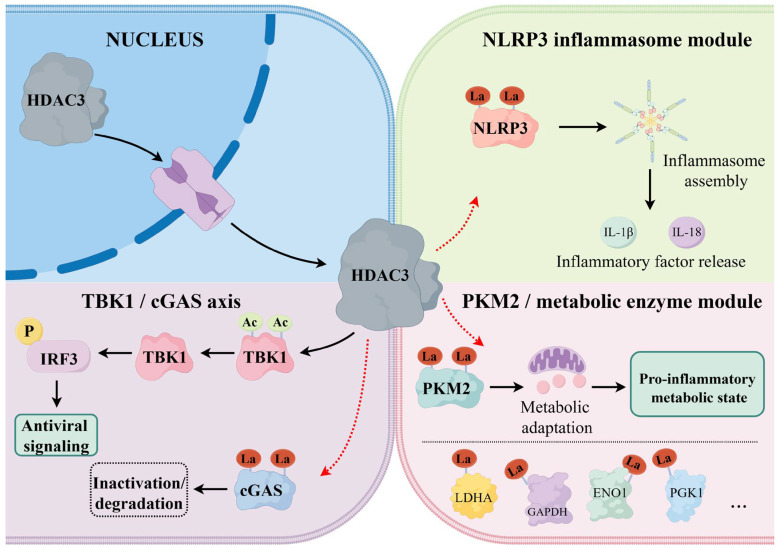
Extra-nuclear HDAC3 and candidate non-histone acylation networks. Note: HDAC3, histone deacetylase 3; TBK1, TANK-binding kinase 1; cGAS, cyclic GMP-AMP synthase; IRF3, interferon regulatory factor 3; NLRP3, NLR family pyrin domain containing 3; IL-1β, interleukin-1 beta; IL-18, interleukin-18; PKM2, pyruvate kinase M2; LDHA, lactate dehydrogenase A; GAPDH, glyceraldehyde-3-phosphate dehydrogenase; ENO1, enolase 1; PGK1, phosphoglycerate kinase 1; Ac, acetylation; La, lactylation; P, phosphorylation. Solid arrows indicate experimentally supported deacetylation events or established downstream relationships. Dashed arrows indicate candidate HDAC3-linked delactylation mechanisms requiring further validation. Dotted-outline boxes denote reported or proposed functional consequences of the modified target rather than direct HDAC3-validated enzymatic events. Colors are used to distinguish different functional modules and do not indicate evidence strength. The figure summarizes established and candidate extra-nuclear functions of HDAC3. Created with Figdraw (ID: TAYSU1e55b).

Overall, the most reasonable current positioning of extra-nuclear HDAC3 is not that of an already established dominant cytoplasmic delactylase, but rather that of a candidate regulatory node with clear spatial plasticity, experimentally supported non-histone deacylation activity, and a surrounding large-scale lactylome background. Together, these lines of evidence define a candidate extra-nuclear action field for HDAC3: TBK1 provides direct deacetylation evidence for HDAC3-mediated control of cytoplasmic immune signaling proteins, cGAS and NLRP3 provide functionally consequential lactylated candidate nodes, and PKM2 together with glycolytic enzyme networks provide a metabolic-enzyme substrate field. Figure 5 summarizes the experimentally supported and candidate relationships within this extra-nuclear HDAC3-associated acylation network.

## 7. Therapeutic Implications and Future Directions: Opportunities and Challenges in the Selective Regulation of HDAC3

HDAC3 is better viewed as an immunometabolic node requiring function-stratified regulation rather than simple catalytic inhibition. Its output depends on NCoR/SMRT complex assembly, subcellular localization, metabolic context, and access to histone and non-histone acylated substrates. Therapeutic strategies should therefore distinguish isoform selectivity, complex dependence, protein-interaction interfaces, and nuclear versus extra-nuclear HDAC3 pools. Compartment-resolved acylation–deacylation maps may further guide mechanism-matched intervention.

### 7.1. Pharmacologic Barriers: The Toxicity and Mechanistic Ambiguity of Pan-HDAC Inhibitors

Broad HDAC inhibition centered on the catalytic pocket played an important role in bringing epigenetic drugs into the clinic, but its limitations have become equally clear. Current reviews and safety analyses indicate that the clinical toxicities of HDAC inhibitors are not isolated events, but display a relatively stable class-effect profile, including hematologic toxicity, gastrointestinal adverse effects, fatigue, and cardiac electrophysiologic risk, with thrombocytopenia and QT interval-related safety concerns being especially notable [91,92,93]. These adverse effects are not simply the result of excessive inhibitory strength, but also reflect cross-inhibition among isoforms, differences in tissue exposure, and off-target pharmacology. Broad inhibition therefore achieves epigenetic modulation at the cost of amplifying systemic toxicity and mechanistic noise.

For HDAC3, this contradiction between broad inhibition and broad toxicity is even more pronounced. HDAC3 cannot be defined solely by its catalytic pocket because its enzymatic activity and biological functions depend strongly on complex assembly and scaffold-like roles. Its efficient enzymatic activity depends on an activated complex jointly stabilized by NCoR/SMRT-DAD and inositol polyphosphates [7,8,20], whereas some of its key biological functions are linked to complex integrity and scaffold-like roles rather than being fully equivalent to deacetylation itself [11,26]. This means that even if conventional small molecules effectively occupy the catalytic site, they may still fail to reproduce the phenotypes associated with genetic deletion or complex dismantling. For HDAC3, catalytic inhibition and functional removal are not the same pharmacologic event, and a natural gap therefore exists between pharmacologic and genetic results.

The advance of lactylation research has added another layer of complexity to this issue. Class I HDACs have been shown to possess delactylase activity, and new biochemical studies further suggest that under specific donor conditions the same enzymatic system may also participate in reverse lactylation [6,83]. In high-lactate or highly glycolytic contexts, the impact of HDAC inhibitors on Kac and Kla networks may therefore no longer reflect simple blockade of a single pathway, but rather a simultaneous perturbation of the balance among multiple acyl modifications. Particularly in metabolically stressed settings such as inflammation or tumors, a given HDAC inhibitor may affect deacetylation, delactylation, non-histone acylation, and complex-occupancy effects at the same time. The outcome is therefore difficult to interpret through the single dimension of reduced HDAC activity.

The core problem facing pan-HDAC inhibitors is thus no longer simply one of potency, but of insufficient resolution across functional layers. Such agents may effectively suppress part of the catalytic output of HDACs, yet remain unable to distinguish among the catalytic function, non-catalytic scaffold function, complex-anchoring function, and spatial relocalization function of HDAC3. For a target with dual-track output and spatial plasticity, pharmacologic intervention without functional stratification easily leads to two consequences: a limited therapeutic window and difficulty in precise mechanistic attribution. This is also an important reason why the same HDAC inhibitory strategy often produces non-identical biological outcomes across different inflammatory or metabolic settings. Drug development around HDAC3 therefore needs to shift from broad inhibition to function-stratified regulation. 

### 7.2. Precision Strategies: Isoform Selectivity, Complex Interfaces, and Targeted Degradation

The key to overcoming these barriers is not to further increase inhibitory potency, but to improve the resolution of intervention. A first step toward higher resolution is isoform selectivity, although this direction itself requires careful interpretation. RGFP966 was widely regarded in early studies as an HDAC3-selective probe and was broadly used in neurobehavioral models [94]. However, later slow-binding kinetic analyses showed that it exerts potent activity against HDAC1, HDAC2, and HDAC3, meaning that its apparent HDAC3 specificity exceeds its true pharmacologic boundary [95]. In the current context, RGFP966 is therefore better described as an early HDAC3-biased probe rather than a rigorously validated highly selective HDAC3 inhibitor.

By contrast, later HDAC3-biased inhibitors and newer chemical scaffolds have provided more useful tools for this direction. HDAC3 inhibition has shown functional benefit in metabolic disease models, including improved glycemic control and insulin secretion in obese diabetic rats [96], as well as reduced islet infiltration and delayed diabetes onset in NOD mice [97]. In addition, hydrazide-based small molecules have been developed as more HDAC3-biased inhibitors and have shown substantial in vitro activity in cancer cell models [98]. These studies support isoform-biased HDAC3 inhibition as a useful experimental and therapeutic direction. Nevertheless, these compounds remain primarily mechanistic probes or candidate tools and have not yet achieved disease-specific, tissue-selective, or metabolically matched regulation of HDAC3.

Importantly, isoform selectivity should not be conflated with complex-state selectivity. RGFP966 and BRD3308 are active-site-directed inhibitors with different degrees of HDAC3 bias, but neither has been demonstrated to selectively distinguish free HDAC3 from NCoR/SMRT-bound HDAC3 in cells. This distinction is mechanistically important because free HDAC3 has low intrinsic catalytic activity, whereas efficient catalysis requires conformational licensing by NCoR/SMRT-DAD and IP_4_ [7,8,20]. Recent biochemical evidence further indicates that HDAC complex composition and inositol-phosphate status can substantially alter inhibitor potency and selectivity [99]. Complex-sensitive HDAC3 pharmacology should therefore currently be regarded as a future design and screening objective rather than an established property of RGFP966, BRD3308, or other available HDAC3-biased inhibitors.

Against this background, a deeper strategy than isoform selectivity is to intervene directly in the complex-dependent regulation of HDAC3. Recent evidence that SMRT anchors the HDAC3 corepressor complex to chromatin further supports the view that complex assembly is itself functionally consequential [85]. Within this framework, targeting the catalytic pocket alone cannot capture the full biological output of HDAC3. Strategies capable of recognizing complex state, altering protein-interaction interfaces, or perturbing complex anchoring may more closely reflect the physiological mode of HDAC3 regulation. Future drug screening should therefore move beyond IC_50_ values obtained with isolated enzymes and incorporate NCoR/SMRT-DAD assembly, IP_4_ status, nucleosomal substrates, and authentic intracellular complex conformations, because these variables can materially influence inhibitor efficacy and selectivity [99].

Targeted protein degradation offers another important intervention route for HDAC3. Unlike active-site inhibition, PROTAC-based molecules can directly reduce HDAC3 protein abundance, thereby affecting both catalytic functions and at least part of its non-catalytic structural roles. The development of HDAC3-specific PROTACs has already demonstrated the feasibility of targeted HDAC3 degradation [100]. Dual HDAC3/8 degraders have also shown value as mechanistic tools for dissecting the contribution of HDAC3/8 to histone acetylation and gene regulation [101]. More specifically, the HDAC3-directed PROTAC P7 can induce HDAC3 degradation in macrophage systems and suppress polarization of M0-like macrophages toward an M1-like pro-inflammatory phenotype, suggesting translational anti-inflammatory potential for degradation-based strategies [102].

Targeted degradation remains an experimental rather than established therapeutic strategy. Its value lies not only in drug development, but also in providing higher-resolution tools for HDAC3 functional stratification, mechanistic dissection, and indication selection. However, degradation does not inherently mean greater safety or precision. Although degradation strategies can cover both catalytic and non-catalytic functions, they may also impose stronger systemic effects, especially given the basal roles of HDAC3 in hepatic metabolism, immune homeostasis, and cell-cycle regulation. For a target as functionally layered as HDAC3, the most valuable molecules may not necessarily be those that remove HDAC3 more completely, but rather those high-quality pharmacologic tools that clearly distinguish among catalytic inhibition, complex perturbation, and protein clearance. Table 2 summarizes the representative HDAC3-related regulatory tools currently available and the boundaries of the evidence supporting them.

### 7.3. Future Course: Building Cytoplasmic Acylation-Deacylation Maps

The next phase of HDAC3 research should not be limited to the continued accumulation of new lactylation sites, but should move toward systematic, compartment-resolved mapping of cytoplasmic acylation and deacylation networks. Current evidence already shows that lactylation is not restricted to histones. Non-histone lactylation has been observed widely across whole-cell protein networks and can directly affect inflammatory amplification, metabolic adaptation, and innate immune signaling output [67]. At the functional level, HMGB1 lactylation is associated with exosomal release [69], PKM2 lactylation can reshape the metabolic program of pro-inflammatory macrophages [71], the AARS1/2-driven lactylation network can directly act on cGAS and suppress DNA sensing [86], and more recent work has moved NLRP3 itself into the category of a protein with direct lactylation and clear functional consequences [87]. Together, these findings indicate that the cytoplasmic lactylome has moved beyond a peripheral extension of histone biology and emerged as a regulatory network with independent mechanistic significance.

For HDAC3, the real priority is not to label all of these proteins as already validated direct substrates, but to establish a stepwise validation framework for its candidate action field. Current evidence supports the idea that HDAC3 regulates TBK1 deacetylation and promotes innate antiviral immunity [89], and also that it participates in antiviral programs in macrophages through the FOXK1-STAT1/2 axis [90]. By contrast, for PKM2, cGAS, and NLRP3, the more cautious current positioning remains that they are key lactylated nodes located within the extra-nuclear candidate action field of HDAC3, rather than substrates already proven to undergo site-specific delactylation by HDAC3. This evidentiary boundary must be maintained, because it directly determines whether future experimental designs will be convincing.

Methodologically, this direction is now becoming tractable. Subcellular fractionation combined with mass spectrometry can already map lactylation distributions across different cellular compartments and help distinguish nuclear, cytoplasmic, and membrane-associated substrate pools [103]. Site-specific lactylation technology based on genetic code expansion is also beginning to move the field from detecting a modification to directly validating the function of an individual site [104]. At the same time, proximity-labeling techniques and live-cell protein-trafficking approaches are making it increasingly feasible to measure protein localization, local interactions, and intercompartmental movement [105,106]. Together, these methods make it possible to move extra-nuclear HDAC3 research from the question of whether it exists to the more informative questions of where it acts, what it contacts, which modifications it processes, and what functional consequences it produces.

A practical validation workflow could begin by integrating compartment-resolved Kla proteomics with HDAC3-centered proximity labeling and HDAC3 perturbation under basal, inflammatory, and high-lactate conditions, thereby prioritizing cytoplasmic proteins that are both spatially accessible to HDAC3 and show HDAC3-dependent changes at defined Kla sites [103,105,106]. Candidate sites could then be examined in HDAC3-deficient or acutely degraded cells followed by rescue with wild-type, catalytically inactive, and full-length HDAC3 constructs engineered for predominant nuclear or cytoplasmic localization. Because alteration of native HDAC3 localization regions may also affect conformational regulation or complex-dependent activation, the localization and catalytic competence of each construct should be verified independently [21,22,81]. Finally, targeted mass spectrometry, site-directed mutagenesis, site-specific lactylation through genetic code expansion, and direct biochemical delactylation assays where feasible could be used to connect individual Kla sites with relevant inflammatory or metabolic outputs [104]. Convergent evidence for spatial access, site-specific regulation, catalytic and compartmental dependence, direct modification removal, and functional rescue would be required before proteins such as cGAS, NLRP3, or PKM2 could be classified as direct cytoplasmic HDAC3 delactylation substrates.

A convincing validation framework should satisfy three conditions. First, it should provide compartmental resolutions and distinguish the nuclear, cytoplasmic, and membrane-associated action fields of HDAC3. Second, it should provide site resolution and determine whether a given Kla or Kac site is regulated in an HDAC3-dependent manner. Third, it should provide functional resolution and directly connect modification changes to biological outputs such as inflammatory cytokine release, interferon responses, inflammasome activation, or altered metabolic flux. Only when the evidence chain is closed across all three levels can extra-nuclear delactylation by HDAC3 move from a plausible hypothesis to a validated and potentially actionable mechanism.

The significance of such maps goes beyond filling in mechanistic detail; they will directly shape indication selection and pharmacologic design for HDAC3-targeted strategies. If the critical pathological processes in a given disease are driven mainly by nuclear chromatin programs, then isoform-selective catalytic inhibition may be appropriate in some settings. By contrast, when pathology is centered more heavily on cytoplasmic nodes such as TBK1, cGAS, NLRP3, or metabolic enzyme networks, the more useful pharmacologic tools will likely be those capable of distinguishing catalytic inhibition from complex disruption, spatial relocalization, and non-catalytic effects. Whether HDAC3 can truly become a precisely actionable immunometabolic target will ultimately depend on resolving its compartment- and substrate-specific functional modules rather than treating it as a single uniform enzyme. Achieving such resolution will be a crucial step in the transition of HDAC3 from a classical deacetylase to a precision therapeutic target.

## 8. Conclusions

HDAC3 may be more appropriately conceptualized not simply as a classical nuclear deacetylase, but as a context-dependent immunometabolic rheostat whose output is shaped by complex state, metabolic and acyl-substrate availability, and compartment-specific substrate access. Efficient catalysis requires licensing by NCoR/SMRT-DAD and IP_4_, whereas some biological functions reflect non-catalytic scaffold and anchoring roles. This dual structural-functional architecture gives HDAC3 remarkable contextual adaptability across distinct inflammatory and metabolic microenvironments. It can maintain transcriptional thresholds and suppress basal noise under homeostatic conditions, yet also be precisely redeployed to specific gene clusters and signaling modules during acute stress, thereby generating a complex regulatory network in which gene licensing, amplitude restriction, and transcriptional redirection coexist.

The emergence of the lysine lactylation paradigm has further reshaped the perceived boundaries of HDAC3 function. Current biochemical evidence is already sufficient to support HDAC3 as an important delactylase within class I HDACs, meaning that HDAC3 faces not a single acetylation landscape, but a dynamic substrate environment jointly shaped by glycolysis, lactate load, and multiple acyl modifications. At the same time, more recent studies suggest that class I HDAC systems may, under specific donor conditions, also participate in reverse lactylation, making HDAC3 more akin to an acyl-processing node with directional plasticity rather than a strictly one-way eraser. Even so, it remains essential to distinguish carefully between what is already supported by direct evidence and what remains a model awaiting validation. The most cautious conclusion at present is that HDAC3 possesses clear delactylase capacity, supported by relatively strong enzymatic evidence; however, whether it undergoes quantifiable catalytic redistribution between acetylated and lactylated substrates in real cellular settings, and whether such redistribution is sufficient to drive phase-specific shifts in inflammatory programs, still requires more direct cellular and in vivo evidence. 

Equally important, a major frontier in HDAC3 research is now moving clearly beyond the nuclear chromatin boundary. The rapid expansion of non-histone lactylation networks means that the cytoplasm is no longer merely a metabolic background, but is becoming a regulatory space in which real functional consequences unfold. cGAS lactylation and its degradation, TBK1 deacetylation-associated amplification of innate immunity, and the antiviral program linked to the FOXK1-STAT1/2 axis together suggest that the extra-nuclear role of HDAC3 is not a scattered set of isolated observations, but may instead correspond to a candidate action field that has not yet been systematically mapped. What deserves emphasis here is not the premature labeling of more proteins as already validated direct substrates, but the recognition that the field has now reached a point at which the relationships among HDAC3 spatial relocalization, extra-nuclear substrate spectra, and acyl-processing modes across different compartments have become critical variables for defining its true mechanistic depth. The most decisive work going forward will no longer be to continue showing that HDAC3 may enter the cytoplasm, but to dissect its nuclear and extra-nuclear functional units more clearly and determine which effects depend on catalytic activity, which depend on complex state, and which primarily reflect spatial localization or non-catalytic scaffold functions.

This shift in understanding also directly changes the translational logic of HDAC3. For a target so strongly dependent on complex state and capable of multilayered functional output, pan-HDAC inhibition is clearly insufficient as a precise therapeutic framework. The class-effect toxicities and mechanistic ambiguities exposed by broad-spectrum inhibitors already show that inhibiting HDAC activity is not equivalent to selectively rewriting the pathological output of HDAC3. The more promising path forward lies in pharmacologic designs with greater resolution across the functional hierarchy of HDAC3: one strategy is complex-state-sensitive selective inhibition, in which enzymatic control is interpreted within authentic complex conformations rather than under free-enzyme conditions; another is intervention directed at protein-interaction interfaces or complex assembly, allowing catalytic and structural functions to be distinguished; and a third is degradation-oriented chemical tools, which can simultaneously cover catalytic output and non-catalytic roles, thereby providing clearer experimental leverage for mechanistic stratification and indication selection. Whether HDAC3 can truly become a precisely actionable immunometabolic target will therefore depend less on proving its importance once again than on resolving, with sufficient depth, how different compartments, different acyl substrates, and different functional layers are related to one another in ways that can support mechanism-matched intervention.

From a broader perspective, the value of HDAC3 research lies not only in explaining the multifunctionality of a single enzyme, but also in providing a representative paradigm for how immunometabolism and epigenetic regulation are coupled at one molecular node. HDAC3 links metabolite abundance to chromatin state, and nuclear transcriptional gating to extra-nuclear signal execution. It also illustrates how complex dependence constrains enzymatic output, while at the same time exposing the limitations of the traditional pharmacologic model of “one target, one function.” For this reason, the most important future direction for HDAC3 is not simply to accumulate more evidence, but to reorganize existing evidence into a truly spatiotemporally resolved systems framework. Only by doing so can HDAC3 move from being an epigenetic molecule of mechanistic importance to becoming an immunometabolic target that can be regulated with genuine precision.

## Figures and Tables

**Table 1:** Evidence spectrum for HDAC3-mediated deacetylation and delactylation.

Evidence Module	Target Activity	Complex/Spatial State	Model and Substrate	Key Findings and Boundary	References
Complex-licensed canonical deacetylation	Histone deacetylation (Kac removal)	Nuclear HDAC3–NCoR/SMRT-DAD complex; IP_4_-stabilized	HDAC3–DAD complexes; acetylated histone-like substrates	DAD–IP_4_ assembly licenses efficient HDAC3 activation	[7,8,18,19,20,81]
Nuclear chromatin gatekeeping	Chromatin deacetylation; transcriptional threshold control	Endogenous HDAC3 in macrophage chromatin	HDAC3-deficient macrophages; LPS and IL-4 programs; chromatin acetylation	Supports selected LPS responses; restrains IL-4-driven alternative activation; context-dependent output	[9,10,46]
Direct delactylase evidence	Histone lysine delactylation (Kla removal)	Purified class I HDACs, including HDAC3	Lactylated peptides, histones, and selected cellular sites	Robust zinc-dependent HDAC3 delactylase activity in vitro	[6,74]
Broad short-chain deacylase capacity	Deacylation beyond acetyl groups	Class I HDACs; biochemical systems	Crotonylated and related short-chain acyl substrates	Broad class I HDAC deacylation; chemical plausibility for HDAC3 delactylation	[15,17]
Supply-side constraint on apparent acyl preference	Acyl-pool-dependent substrate selection	HDAC3 complexes in metabolically altered nuclear environments	Acetyl-CoA and lactoyl-CoA pools; compartmentalized mixed acyl substrates	Preference shaped by metabolite supply, compartment, isomer, and substrate burden	[16,63,75,76,77,82]
Reversibility and evidence boundary	Context-dependent acyl exchange	Class I HDACs under varied donor–acceptor conditions	Reverse-lactylation assays integrated with delactylase data	Biochemical support for context-dependent acyl processing; cellular Kac–Kla redistribution unproven	[6,83]
Extra-nuclear candidate action field	Candidate extra-nuclear non-histone deacylation	Extra-nuclear HDAC3; cytosolic or membrane-associated compartments	Non-histone lactylomes; immune sensors; metabolic enzymes	Plausible extra-nuclear substrate field; direct cytosolic HDAC3 delactylation unproven	[12,21,22,23,24,66,67,68,69,70,71,72,84,85,86,87,88,89,90]

Note: HDAC3, histone deacetylase 3; HDAC1–3, class I histone deacetylases 1–3; Kac, lysine acetylation (or acetyl-lysine); Kla, lysine lactylation (or lactyl-lysine); NCoR, nuclear receptor corepressor; SMRT, silencing mediator for retinoid and thyroid hormone receptors; DAD, deacetylase activation domain; IP_4_, inositol 1,4,5,6-tetrakisphosphate; Acetyl-CoA, acetyl coenzyme A; lactoyl-CoA, lactyl coenzyme A. In this table, Kac–Kla refers to the relative balance between lysine acetylation and lysine lactylation. IP_4_ and Ins(1,4,5,6)P_4_ refer to the same inositol phosphate cofactor.

**Table 2:** Representative pharmacologic and chemical tools related to HDAC3 targeting and regulation.

Agent	Pharmacologic Class	HDAC3-Relevant Mechanism	Selectivity/Evidence Boundary	Representative Biological Context	Key Takeaway	References
Lactate	Endogenous metabolism-linked regulator	Broad HDAC inhibition; histone hyperacetylation	Not HDAC3-selective; metabolic context regulator	Glycolytic or high-lactate cellular contexts	Models lactate-rich HDAC regulation; unsuitable for selective HDAC3 modulation	[4]
Ins(1,4,5,6)P_4_/inositol phosphate	Endogenous allosteric cofactor/complex regulator	Stabilization of the HDAC3–DAD interface	Allosteric cofactor, not inhibitor; complex-dependent effects	Structural biochemistry and complex pharmacology	Frames complex-dependent HDAC3 pharmacology; not a therapeutic agent	[8,20,99]
RGFP966	Benzamide-based class I HDAC inhibitor/slow-binding chemical probe	Active-site inhibition of class I HDACs	HDAC3-biased, not HDAC3-specific; inhibits HDAC1–3	Behavioral and inflammatory model studies	Useful mechanistic probe; HDAC3-specific attribution requires caution	[94,95]
BRD3308	HDAC3-biased/isoform-selective inhibitor	HDAC3-biased catalytic inhibition	Reported HDAC3-selective inhibitor; no demonstrated complex-state selectivity	Diabetic rat and NOD mouse models	Improved glycemia and insulin secretion; reduced islet infiltration; protected against diabetes	[96,97]
Compound 4e	Hydrazide-based HDAC3 inhibitor	Potent direct inhibition of HDAC3	HDAC3 IC_50_ = 15.41 nM; ≥18-fold selectivity; cell-based evidence	Cancer cell-line systems	Promising selective scaffold; no in vivo validation	[98]
XZ9002	HDAC3-directed PROTAC degrader	Induces targeted degradation of HDAC3 protein	Reported first-in-class HDAC3-specific PROTAC; breast cancer cell evidence	Breast cancer cell lines	HDAC3 degradation; antiproliferative effects beyond catalytic inhibition	[100]
P7	HDAC3-directed PROTAC degrader	Induces near-complete HDAC3 degradation in macrophage systems	Selective HDAC3 PROTAC; macrophage evidence	THP-1 cells and human primary macrophages	Reduced pro-inflammatory cytokines; blocked M0-like to M1-like polarization; anti-inflammatory potential	[102]
YX968	Dual-target PROTAC degrader	Simultaneous degradation of HDAC3 and HDAC8	Dual-target degrader; not HDAC3-specific; primarily a mechanistic tool	Chromatin acetylation and gene regulation studies	Dissects HDAC3/8 chromatin functions; unsuitable for HDAC3-specific attribution	[101]

Note: HDAC3, histone deacetylase 3; HDAC1/2/3, histone deacetylases 1, 2, and 3; NCoR, nuclear receptor corepressor; SMRT, silencing mediator for retinoid and thyroid hormone receptors; DAD, deacetylase activation domain; IP_4_, inositol 1,4,5,6-tetrakisphosphate; PROTAC, proteolysis-targeting chimera; IC50, half maximal inhibitory concentration; NOD, nonobese diabetic; THP-1, human monocytic leukemia cell line.

## Data Availability

No new data were created or analyzed in this study.
